# Measuring mental wellbeing in clinical and non-clinical adolescents using the COMPAS-W Wellbeing Scale

**DOI:** 10.3389/fpsyt.2024.1333828

**Published:** 2024-06-27

**Authors:** Janine R. Lam, Haeme R. P. Park, Justine M. Gatt

**Affiliations:** ^1^ Centre for Wellbeing, Resilience and Recovery, Neuroscience Research Australia, Sydney, NSW, Australia; ^2^ School of Psychology, University of New South Wales, Sydney, NSW, Australia

**Keywords:** mental health, well-being, adolescence, psychiatric disorders, developmental disorders, psychometric testing, reliability, validity

## Abstract

**Introduction:**

Adolescence is a key period of vulnerability for poor mental health as the brain is still developing and may be more sensitive to the negative impacts of stress and adversity. Unfortunately, few measures comprehensively assess wellbeing in adolescents.

**Methods:**

The 26-item COMPAS-W Wellbeing Scale for adults was validated in a sample of 1,078 adolescents aged 13–17 years old (51.67% male, 79.13% non-clinical vs 20.87% psychiatric or developmental clinical cases). The six COMPAS-W sub-scales and total scale were examined in this sample using second-order confirmatory factor analysis, and psychometric testing.

**Results:**

The 23-item COMPAS-W demonstrated the best fit for this sample according to goodness-of-fit indices (χ*^2^* (220, 1078) = 1439.395, *p* < 0.001, *CFI* = 0.893, *TLI* = 0.877, *RMSEA* = 0.070, *SRMR* = 0.095). Internal reliability for the confirmed 23-item COMPAS-W model was run for the total scale (*α* = 0.912) and sub-scales (Composure, *α* = 0.735; Own-worth, *α* = 0.601; Mastery, *α* = 0.757; Positivity, *α* = 0.721; Achievement, *α* = 0.827; and Satisfaction, *α* = 0.867). Test-retest reliability over 6 weeks was also good for the total scale at *r* = 0.845 and the sub-scales: Composure (*r* = 0.754), Own-worth (*r* = 0.743), Mastery (*r* = 0.715), Positivity (*r* = 0.750), Achievement (*r* = 0.750), and Satisfaction (*r* = 0.812). Compared with non-clinical participants’ wellbeing (*M* = 90.375, *SE* = 0.400), those with clinical diagnoses reported lower wellbeing, both for those with developmental diagnoses (*M* = 85.088, *SE* = 1.188), or psychiatric diagnoses (*M* = 78.189, *SE* = 1.758), or combined developmental and psychiatric diagnoses (*M* = 77.079, *SE* = 2.116). Yet, when wellbeing category scores were considered by diagnosis group, both non-clinical and clinical groups demonstrated incidence across all three categories of languishing, moderate and flourishing wellbeing, in support of the dual-continua model of mental health. On average, younger adolescents’ (13–14 years) wellbeing did not differ from older adolescents’ (15–17 years) wellbeing; however, for sex, males scored 1.731 points significantly higher in wellbeing compared with females (*p* = 0.028); and American participants scored 3.042 points significantly higher in wellbeing compared with Australian participants (*p* < 0.001).

**Discussion:**

In conclusion, the 23-item COMPAS-W is a reliable measure of wellbeing for adolescents, both for those with and without developmental and psychiatric diagnoses.

## 1 Introduction

Adolescence is a key period of vulnerability for poor mental health as the brain is still developing and may be more sensitive to the negative impacts of stress and adversity (1). In adolescents, reported rates of anxiety and depression range from 7.8 to 19.3% in Western countries, and rates of developmental difficulties (such as ADHD, autism and dyslexia) range from 2.4% to 8.7% (2, 3). Fortunately, in addition to risk, the period of adolescence holds a window of opportunity in brain development to facilitate a flourishing state of mental wellbeing which may reduce the risk of mental illness (1). Research has found that while adolescents languishing in wellbeing report an average of 10.4 depressive symptoms, flourishing adolescents report a much lower average of 1.4 depressive symptoms (4).

To measure wellbeing, early leaders in wellbeing research focused on measuring hedonic constructs otherwise known as ‘subjective wellbeing’ (5, 6) to evaluate short-term happiness (7, 8) (positive and negative affect) (9) and long-term life satisfaction (7, 10, 11). In adolescents, example measures include assessing how satisfied the adolescents are with their lives, or quality of life. For example, the Student’s Life Satisfaction Scale (12) assesses life satisfaction in those aged 7–14 years. However, only life satisfaction is assessed and no other constructs of subjective wellbeing are included in the measurement. The Multidimensional Students’ Life Satisfaction Scale (13) validated for those in grades 9–12 (14–18 years) goes a step further to assess life satisfaction across the domains of family, friends, school, and living environment, but again, only one aspect of subjective wellbeing is assessed. Other theorists have since argued wellbeing should be viewed primarily through a eudaimonic lens or a ‘psychological wellbeing’ perspective which focuses on positive functioning (11, 14–16). For instance, Ryff (2014)’s measure encompasses six of these dimensions: purpose in life, personal growth, environmental mastery, autonomy, positive relations, and self-acceptance.

Although the concepts of subjective and psychological wellbeing are distinct, they are also related. In Keyes et al.'s (17) study, positive correlations were found between all subjective and psychological wellbeing scales. Further, although their measures of subjective and psychological wellbeing comprised of distinct factors, they were also connected by some overlapping indicators. Therefore, both subjective and psychological wellbeing are crucial to consider when measuring mental wellbeing. In an effort to help people flourish, Seligman has integrated both subjective (positive emotion and engagement) and psychological dimensions (positive relationships, meaning, and achievement) into his PERMA theory of wellbeing (18) which provides a comprehensive account of all subconstructs to consider when measuring wellbeing. *Positive emotion* incorporates happiness and life satisfaction; *engagement* taps into Csikszentmihalyi and LeFevre’s concept of ‘flow’, ‘living in the present moment’, being completely absorbed in a task (19); *positive relationships* with others helps one to feel supported and valued; *meaning* involves belonging to or serving something greater than oneself or ‘having a purpose in life’; and *achievement* (or mastery or competence) is defined as ‘a sense of achievement’ when mastering a goal, but also as having the motivation, perseverance, or passion to achieve goals (which may be intrinsic or external).

Several researchers have attempted to include subjective and psychological dimensions in adolescent measures, many of which include Seligman’s proposed subconstructs. Keyes created the Child Development Supplement (CDS) for adolescents aged 12–18 years in the American longitudinal household survey [Panel Study of Income Dynamics (PSID) (20)]. This measure included Keyes’ own diagnostic criteria for a comprehensive measure of subjective wellbeing (positive affect, avowed happiness, and avowed life satisfaction); psychological wellbeing (self-acceptance, personal growth, purpose in life, environmental mastery, and autonomy); and social wellbeing (which could also be considered as psychological wellbeing as per Ryff’s wellbeing scale (6), social acceptance, social actualization, social contribution, social coherence, positive relations with others, and social integration) (21). The next iteration, CDS II, was psychometrically evaluated. A three-factor model (subjective wellbeing, psychological wellbeing, and social wellbeing) showed good model fit (*Goodness of Fit Index* = 0.93, *Critical N* = 186; *Root Mean Square Error of Approximation* = 0.09, *Akaike Information Criterion* = 672), with modestly strong correlations between these factors (*r* = 0.57–0.71), and reliability for the factors ranging from *α* = 0.78–0.84. These results show that wellbeing amongst adolescents is characterised by a complex blend of both subjective and psychological wellbeing, rather than any single dimension. The version of the CDS available to researchers is the Mental Health Continuum-Short Form (MHC-SF). Unfortunately, the psychometric properties referenced for the MHC-SF are actually not for adolescents – but only point to validation studies in adult samples or CDS II adolescent validation studies which do not include the same items (e.g., purpose in life and self-esteem are excluded in the CDS II). In addition, test-retest reliability was not conducted (11), therefore it is unclear whether the MHC-SF measure is valid for adolescents (at least for English speakers).

Several others have integrated selected facets of subjective and psychological wellbeing into combined wellbeing measures. One example is the Warwick-Edinburgh Mental Well-being Scale (22) and the scales which have been developed from it. Tennant et al. originally developed the Warwick Edinburgh Mental Well-Being Scale and tested it in late adolescent (16 years+) and population samples, and it included measures of subjective wellbeing (feelings of optimism, cheerfulness, and relaxation) and psychological wellbeing in terms of interpersonal relationships and positive functioning (energy, clear thinking, self-acceptance, personal development, competence, and autonomy) (22) but did not include Seligman’s dimensions of engagement and meaning. Subsequently, Clarke et al. (23) tested the Warwick Edinburgh Mental Well-Being Scale in adolescents 14–16 years, with internal reliability shown to be high (*α* = 0.87), however test-retest reliability estimates showed scope for improvement (*ICC* = 0.66 (*95% CI* [0.59; 0.72] *n* = 212)) given recommended reliability thresholds are 0.70 and above (24, 25). Later a shortened version, the Short Warwick Edinburgh Mental Well-Being Scale (26) was validated with 829 adolescents aged 13–16 years. Although the model demonstrated good fit of the scale (*χ^2^* (*df* = 13) = 30.75, *p* = 0.004, *CMIN/DF ratio* = 2.37, *CFI* = 0.99, *TLI* = 0.99, *RMSEA* = 0.040 [*90% CI*:.022,.059], *SRMR* = .020) and good internal reliability (*α* = 0.87), there was only support for metric invariance but not scalar invariance for sex, indicating that model factor loadings but not intercepts were the same for males and females; thus, the measure cannot be used to provide unbiased comparisons of wellbeing by sex. The study was also cross-sectional and test-retest reliability was not reported. In another study, Liddle & Carter (27) used the components of the Warwick-Edinburgh Mental Well-being Scale to build the Stirling Children’s Well-being Scale, a wellbeing measure for a sample of 8–15 year old young people. Although internal reliability (*α* = 0.847), test-retest reliability (*r* = 0.752), and construct validity (*r* = 0.694–0.742) results suggested the scale was a reliable measure of wellbeing in children, other fundamental validity procedures such as confirmatory factor analysis were not considered or reported.

Other wellbeing scales have been developed specifically for the school context. McLellan and Steward’s School Wellbeing Scale (28) for children (7–16 years old) was inspired by Huppert and So’s (29) use of both subjective and psychological wellbeing in their adult indicators for social policy study. However, the School Wellbeing Scale itself includes just three psychological wellbeing measures (self-acceptance, environmental mastery, and positive relations with others) in addition to subjective wellbeing measures (positive affect, avowed happiness, and avowed life satisfaction), again not including any assessment of Seligman’s suggested dimensions of meaning and engagement. And while the measure was designed for the school context, its applicability beyond this environment is undetermined. In addition, the authors acknowledge model fit could be improved and other core tests such as internal reliability, test-retest reliability, and concurrent validity analyses were not conducted. Therefore, the measure’s validity has not been established.

Despite the contribution of the above studies to wellbeing research in young people, they all have their limitations. For instance, all fall short of comprehensively measuring wellbeing and all of its subcomponents. Almost all offer limited insight into specific aspects of adolescents’ wellbeing and how they may be affected by different factors; that is, no information on sub-factors are typically reported, with only total scores available (for all but the School Wellbeing Scale). The integrity of some of the different scale properties remain untested, and several of the scales were validated with mixed samples of adolescents and adults (22), or adolescents and children (27, 28, 30). However, the adolescent phase of life comprises a unique developmental period which is different to that of children or adults, which could mean adolescents understand or interpret scale items differently to children or adults, or that the scale items may be less relevant to them during their specific developmental phase. Therefore, it is important to test scale performance exclusively in an adolescent cohort.

The COMPAS-W wellbeing scale may provide a way forward. The COMPAS-W is a comprehensive measure of subjective wellbeing (hedonia) and psychological wellbeing (eudaimonia), and adds an additional dimension of Composure to evaluate regulation processes during stress (useful if measuring resilience to adversity is the objective). The COMPAS-W structure has been defined by exploratory factor analysis (EFA) and confirmed by confirmatory factor analysis (CFA) within an adult population (31), and is comprised of 26-items and six sub-scales (31): **Composure**, competency and adaptability in stressful situations; **Own-worth**, autonomy and independent self-worth; **Mastery**, self-confidence and perceived control over one’s environment; **Positivity**, optimism and positive outlook; **Achievement**, goal orientation and striving; and **Satisfaction**, satisfaction with life, health, work, personal relationships, and emotions. The scale has been validated in a sample of 1,669 healthy adult twins (18–61 years) with good demonstrated internal reliability (*a* = 0.84) and test-retest reliability (*r* = 0.82) (31). Good internal reliability of the total COMPAS-W score has also been demonstrated in a sample of 12–17-year-old young people from Australia, Canada, China, and New Zealand as part of an international cross-sectional study on trauma, resilience, and mental health (*α* = 0.824–0.900, *n*= 194), but no other psychometric testing has been performed for adolescents as yet (32).

These results indicate potential use of COMPAS-W as a measure of wellbeing for adolescents, and therefore the purpose of the current study is to expand on this initial testing of COMPAS-W in a larger sample of adolescents aged 13–17 years who vary in mental health status (non-clinical and clinical young people), and to evaluate its reliability and validity using more in-depth psychometric analysis. We will employ several psychometric approaches to validate the COMPAS-W in adolescents. First, we will use CFA to verify whether the existing COMPAS-W factor structure established in adults (31) is appropriate for adolescents. We will also establish the internal consistency and test-retest reliability of the total scale and sub-scales over six weeks.

To evaluate how valid COMPAS-W is in measuring adolescent wellbeing, we will assess how closely COMPAS-W scores are related to similar wellbeing scales using correlation analysis. COMPAS-W is negatively correlated with the Depression and Anxiety Stress Scale (DASS-21) (which measures psychological distress) in adults (31, 33) and we would expect COMPAS-W to be similarly correlated in adolescents with other negative mental health and behavioural measures relevant to this developmental period such as the Strengths and Difficulties Questionnaire (SDQ) (34) (behavioural problems), negative affect from the Positive and Negative Affect Schedule (PANAS) (35), the Cyberbullying Scale (36) (cyber-victimisation), and the De Jong Gierveld Loneliness Scale (37) (loneliness), particularly as wellbeing has been found to have a strong inverse genetic correlation with loneliness (38). Conversely, we expect COMPAS-W to be positively correlated with psychological scales measuring positive affect (PANAS), resiliency resources [Resilience Research Centre Adult Resilience Measure (RRC-ARM)] (39), and approach coping (The Brief Coping Orientation to Problems Experienced [Brief-COPE (40)] which is an adaptive form of coping during stress (41)).

Given wellbeing has been shown to differ across key demographics such as age, sex, and country, we will also seek to examine adolescent wellbeing by these variables. With regards to age, some evidence exists for a decline in wellbeing as children grow older. For instance, Renshaw and Bolognino’s (42) Psychological Wellbeing and Distress Screener results indicated lower wellbeing as grade level increased (10–16 years). Further, Keyes’ (4) reported the prevalence of a state of flourishing to be higher for those 12–14 years compared to those 15–18 years. In contrast, no differences in wellbeing scores by age were found in Hunter et al.’s study in 13–16 year old young people (26) or Clarke et al.’s study of adolescents aged 13–16 years and over (23). Given these conflicting associations between wellbeing and age, further investigation is required.

In addition, wellbeing may vary by sex. Tennant et al’s (22) Warwick Edinburgh Mental Well-Being Scale study in student and population samples aged 16 years and over reported wellbeing to be higher in men than in women. Similarly, Clarke et al. (23) found wellbeing to be higher in boys than girls in those aged 13–16 years. Butler et al. (43) also found females to have significantly lower mental wellbeing compared with males 8–15 years old. Given the trend for females to experience lower wellbeing than males, sex differences in wellbeing will also be examined for this sample.

Lastly, differences in wellbeing by country of residence have also been reported. The Mental State of the World in 2022 report found that in 2021, American residents had better mental wellbeing than Australian residents (44). The average Mental Health Quotient score (capturing clinical symptoms and healthy functioning) for the USA was 67.9 and for Australia 54.4. Further, Cosma et al’s research indicated life satisfaction amongst adolescents varied across countries (45) and Marquez et al. (46) found life satisfaction scores to vary in 15-year-old adolescents across England, Scotland, Japan, Northern Ireland, Wales, USA, Ireland, and France. These findings indicate cultural differences in wellbeing may exist, thus we will examine country differences in wellbeing in our sample.

In terms of mental wellbeing and mental illness, young people may concurrently experience high or low levels of either outcome at the same time; that is, the presence of diagnosed illness does not necessarily mean the absence of wellbeing, and vice versa. Keyes’ work demonstrated dual-continua model of mental health and mental illness in adolescents (47) while showing separate latent factors of mental wellbeing and mental ill health to be negatively correlated (*r* = -.68). Greenspoon and Saklofske’s dual-continua model in children aged 8–12 years (48) also showed that wellbeing and psychopathology are not opposing poles of a continuum, but that children may experience high wellbeing and psychopathology at the same time, or a range of other combinations of low/high wellbeing and presence/absence of psychopathology concurrently. Therefore, we will also compare wellbeing in non-clinical and clinical participants in our sample to examine prevalence differences in different groupings.

In summary, the aims of this study were (i) to confirm the factor structure, and establish internal and test-retest reliability of COMPAS-W for the total scale and sub-scales in a large adolescent cohort; (ii) to determine criterion validity by correlating the total COMPAS-W wellbeing scores with positive and negative health behaviours relevant to the adolescent period including measures of depression and anxiety symptoms (DASS-21), behavioural problems (SDQ), resiliency resources (RRC-ARM), coping styles (Brief-COPE), loneliness (De Jong Gierveld Loneliness Scale), cyberbullying (the Cyberbullying Scale), and positive and negative affect (PANAS); (iii) to examine whether mental wellbeing in adolescence differs by key demographic factors such as age, sex, and country of residence; and (iv) to determine whether mental wellbeing differs for adolescents who have a developmental diagnosis, psychiatric diagnosis, or both developmental and psychiatric diagnoses, relative to non-clinical samples, and the degree of difference between these groups.

## 2 Materials and methods

### 2.1 Participants

The current study included 1,078 adolescents aged 13 to 17 years tested across Australia and the United States. The data for the current study was derived from a larger study of 2,580 adolescents and young adults aged 13 to 25 years which aimed to examine the impact of social media use on wellbeing (41). Participants were recruited through a third party, Qualtrics, who sent their database members our online Qualtrics survey. Each participant completed a battery of surveys which assessed mental health and wellbeing as well as other health and lifestyle questions. Inclusion criteria required participants to speak English as a primary language. Baseline data was collected from May 1st to June 10th, 2020. Participants were required to complete four 20-minute surveys, six weeks apart, over six months. For each survey, participants were given an incentive of 10 points or gift vouchers. Digital fingerprint technology was used by the Qualtrics recruitment team to avoid duplication and provide validity of the data collected remotely. Deduplication was also used to ensure reliability and integrity of the data.

The current study aimed to validate the COMPAS-W wellbeing scale for participants aged 13–17 years only to focus on the adolescent period. Data from COMPAS-W as well as other criterion measures (e.g., SDQ) and demographic data from the first two timepoints were used. Validation of COMPAS-W included reliability and confirmatory factor analyses.

#### 2.1.1 Diagnosis group

To examine wellbeing across the mental health spectrum, we categorised participants into the following groups: non-clinical (no diagnoses reported); ‘developmental diagnoses’ (answered ‘yes’ to the question *‘Have you ever experienced or been diagnosed with a learning or developmental disorder, such as attention deficit hyperactivity disorder (ADHD), conduct disorder, or learning difficulties?’*); ‘psychiatric diagnoses’ (answered ‘yes’ to the question *‘Have you ever been diagnosed with a psychiatric or psychological condition? (e.g. eating disorder, anxiety disorder, depression, social phobia, PTSD)?’*); and ‘both developmental and psychiatric diagnoses’ (answered ‘yes’ to both questions *‘Have you ever experienced or been diagnosed with a learning or developmental disorder, such as attention deficit hyperactivity disorder (ADHD), conduct disorder, or learning difficulties?’* and *‘Have you ever been diagnosed with a psychiatric or psychological condition? (e.g. eating disorder, anxiety disorder, depression, social phobia, PTSD)?’*). All groups were mutually exclusive.

### 2.2 Ethical standards

The study was approved by the Human Research Ethics Committee of the University of New South Wales (UNSW), Sydney, Australia (HC200150). The studies were conducted in accordance with the local legislation and institutional requirements. Written informed consent was required from participants over the age of 18 years. For those participants under the age of 18 years, written informed consent was initially required from the participant's parent/legal guardian, followed by the participant's implied consent on the participant's completion of the survey.

### 2.3 Measures

Data used for this study included measures of mental wellbeing, mental health, coping, resilience resources, loneliness, mood, and bullying measures, as well as demographic measures.

#### 2.3.1 Mental wellbeing: COMPAS-W

COMPAS-W is a comprehensive mental wellbeing measure, measuring both subjective (hedonic) and psychological (eudaimonic) aspects of wellbeing. This measure provides a total wellbeing score and six sub-scale scores: Composure, Own-worth, Mastery, Positivity, Achievement, and Satisfaction (31). COMPAS-W comprises 26-items which are answered on a five-point scale (1 = strongly disagree, 2 = disagree, 3 = neutral, 4 = agree, 5 = strongly agree). The items were treated as metric variables, with all 26 items summed to calculate the total score. Higher scores indicate better wellbeing. Good internal reliability (*a* = 0.883) has been demonstrated in a previous sample of 194 12–17 year old adolescents (32).

The following scales were used as criterion measures to validate COMPAS-W in this adolescent sample, most of which have been previously used in other adolescent cohorts. For instance, the SDQ, DASS-21, Brief-COPE, and Cyberbullying Scale have been used in previous studies involving adolescent samples (32, 36, 41) with two of these involving COMPAS-W (32, 41). The RRC-ARM was adapted from the Child and Youth Resilience Measure (39), and the De Jong Gierveld Loneliness Scale has been tested on a sample of early adolescents (49), as has the PANAS (50). Reliabilities of these scales for the current sample have been provided below.

#### 2.3.2 Depression Anxiety Stress Scale (DASS-21)

The DASS-21 is a short form of Lovibond and Lovibond’s (33) 42-item adult measure of psychological distress and is used in research and clinical practice. The measure includes 21 items which form three sub-scales: depression, anxiety, and stress (33). Examples of items include *“I felt down-hearted and blue”* and *“I felt that life was meaningless”*. Items are answered on a four-point scale: 0 (did not apply to me at all) to 3 (very much or most of the time). To calculate the total score, all sub-scales were added together and then multiplied by two in order to be interpreted on the standard 42-item scale. Higher scores indicate greater severity of distress. The DASS-21 demonstrated high internal reliability in our sample (*α* = 0.977).

#### 2.3.3 Strengths and Difficulties Questionnaire (SDQ)

The SDQ is a commonly used behavioural screening and treatment outcome questionnaire (34), developed originally for those aged 3–16 years old. The SDQ comprises 25 positive and negative items which are answered on a three-point scale: 0 (not true) to 2 (certainly true). Examples include *“I am helpful if someone is hurt, upset or feeling ill”* and *“I think before I do things”*. Five sub-scales for the SDQ have been identified: emotional symptoms, conduct problems, hyperactivity-inattention, peer problems, and prosocial behaviour. The first four sub-scales are summed to produce a total difficulties score. A higher total difficulties score indicates greater difficulties than a lower score. The scale showed high internal reliability in our sample (*α* = 0.834).

#### 2.3.4 Resilience resources: the Resilience Research Centre Adult Resilience Measure (RRC-ARM)

The RRC-ARM contains 28 items that measure resilience resources (39). Examples of items include *“I am aware of my own strengths”* and *“I feel supported by my friends”*. Responses range from 1 (Not at all) to 5 (A lot). The total RRC-ARM score is calculated by summing all individual item scores together. Higher scores indicate higher resilience resources. The five sub-scales represent the contextual resources that support the path to resilience. The total scale has high internal reliability in our sample (*α* = 0.950).

#### 2.3.5 De Jong Gierveld Loneliness Scale

The adult 6-item De Jong Gierveld Loneliness Scale is a brief measure of overall loneliness, including positive and negatively worded items (37). Example items include *“There are enough people I feel close to”* and *“I miss having people around”*. Positive item responses are scored as Yes = 0, More or Less = 1, and No = 1. Negative items are reverse scored as Yes = 1, More or Less = 1, and No = 0. The total loneliness score is calculated by adding together all six items. Lower scores indicate the respondent is not lonely and higher scores indicate the respondent is very lonely. The scale shows acceptable internal reliability in our sample (*α* = 0.705).

#### 2.3.6 The Positive and Negative Affect Schedule or (PANAS)

The PANAS is comprised of 20 items that measure positive and negative mood, consisting of 10 mood items each (35). Examples of items are *“Excited”* (positive) and *“Afraid”* (negative). Each item is answered on a five-point scale: 1 (Very slightly or not at all) to 5 (Extremely). A higher positive affect score indicates more positive affect, while a lower negative score indicates less negative affect. The PANAS scales have high internal reliability in our sample (positive scale, *α* = 0.925, negative scale *α* = 0.939).

#### 2.3.7 The Cyberbullying Scale

The children’s Cyberbullying Scale is a measure of the experience of being bullied through electronic media (36). Three items from the Cyberbullying Scale (36) were used in this study to measure the experience of cyber-victimisation: *“How often does another kid say something mean to you (such as calling you names or making fun of you) in a text message or online?”*; *“How often does another kid put you down online by sending or posting cruel gossip, rumours, or something else hurtful?”*; and *“How often does another kid pretend to be you and send or post something that damages your reputation or friendships?”*. Participants answer the items on a five-point scale 1 (Never) to 5 (All the time). The total score is calculated by summing all items together, with higher scores indicating a higher frequency of bullying. The Cyberbullying Scale showed high internal reliability in our sample (*α* = 0.904).

#### 2.3.8 The Brief Coping Orientation to Problems Experienced (Brief-COPE)

The Brief-COPE (40) is the short-form of the Coping Orientation to Problems Experienced (COPE) inventory which assesses various ways of coping with stressful life events (51). It contains 28 items and 14 sub-scales which can be combined to evaluate two higher-order sub-scales representing Active or Disengaged coping strategies. Examples of items include *“I’ve been trying to come up with a strategy about what to do”* (Active Coping) and *“I’ve been giving up trying to deal with it”* (Disengaged Coping). Responses range from 0 (I haven’t been doing this at all) to 4 (I’ve been doing this a lot). Higher Active Coping scores indicate more effective coping and higher Disengaged Coping scores represent less effective coping. Both sub-scales have high internal reliability in our sample (Active Coping *α* = 0.935 and Disengaged Coping *α* = 0.942).

### 2.4 Analyses

Data cleaning included a number of steps. Two test questions (both reading “Please select ‘strongly agree’ for this question”) were included in the survey battery to check if responses for each participant were legitimate. If a participant provided an answer that was not ‘strongly agree’, the responses for that participant were removed from the dataset. Missing data on the COMPAS-W items was replaced with the series mean for cases when < 10% of the data was missing.

#### 2.4.1 Confirmatory factor analyses (CFA), reliability, and validity

Internal consistency was initially assessed using Cronbach’s alpha with recommended thresholds being ≥ 0.70 (52).

As our previous work used EFA and CFA to establish the six-factor structure of the COMPAS-W in adults (31), here we ran a CFA to confirm item and factor structure fit for the same scale and sub-scales in adolescents. This CFA model consisted of a hierarchical structure with the six sub-scales reflected by six factors at the base level, and then each of these six factors (i.e., COMPAS-W sub-scales) loading onto a higher-order common factor of ‘total wellbeing’ consistent with a total summed COMPAS-W score.

We conducted the CFA in R using the *Lavaan* package (0.6.16) (53) with the full dataset of COMPAS-W responses (*n* = 1,078) as input. We used default settings where factor variances and covariances were automatically specified for all latent variables and the variance of latent variables was not fixed. All first order factors were correlated. The data was non-normal (univariate and multivariate) and therefore robust maximum likelihood estimation (MLR) was used. MLR is suitable for complete and incomplete, non-normal data (53). The latent variables were not scaled.

The best fitting model was initially guided by the overall fit statistics: chi-square (χ*^2^*), Comparative Fit Index (*CFI*, acceptable fit ≥ 0.95), Tucker–Lewis index (*TLI*, good fit ≥ 0.95), Root Mean Square Error of Approximation (*RMSEA*, close fit ≤ 0.05–0.06), and Standardized Root Mean Square Residual (*SRMR*, acceptable fit ≤ 0.08). In addition, all items needed to load significantly on to their respective factors. We achieved best fit by removing the weakest items from the model. In factor analysis, items with low factor loadings contribute less variation to the latent variables, therefore they may be deleted while retaining items with higher factor loadings in the model (52). We therefore deleted item(s) with the weakest factor loadings (insignificant and/or negative) in the original model and then compared the resulting model to the previous nested model to see if the new model’s fit statistics and item loadings improved. We then retained this new structure and again looked at potential item(s) to delete to refine the model further, taking into consideration the overall fit statistics above. During this process, we also considered the qualitative contribution of the scale items before proceeding with item deletion (that is, whether a similarly worded item was already in the scale and so its removal would not impact scale integrity), and whether the item was appropriate for adolescent groups. We repeated this process iteratively until we achieved the best fitting model.

Once the best fitting model was ascertained, internal reliability was re-assessed for the final item set. We also assessed ‘composite reliability’ which accounts for the multiple factors and factor loading weights in the CFA model when calculating the reliability estimates (52), although we caution against citing these estimates when comparing to other wellbeing scales in the literature as these estimates are often not calculated or reported in other studies. Test-retest reliability for the total scale and sub-scales was evaluated over a six-week period using intra-class correlation coefficients of scores at the two time points.

We performed invariance testing (54) by sex and country to check whether the final factor structure (configural invariance), factor loadings (metric invariance), or factor loadings and intercepts (scalar invariance) were equal across groups, or whether they varied for the different groups. Therefore, to test for configural invariance, the same factor structure was imposed on all groups; to test for metric invariance, the factor loadings of each group were constrained to be equal; and to test for scalar invariance, loadings and intercepts of each group were constrained to be equal. The significance of the models was ascertained by comparing change in the overall fit indices between the nested models.

Criterion validity was determined by correlating the COMPAS-W total score with the mental health, coping, and bullying measures: DASS-21, SDQ, RRC-ARM, Brief-COPE, 6-item De Jong Gierveld Loneliness Scale, the Cyberbullying Scale, and PANAS. Given we were validating the scale for adolescents, we also included the SDQ sub-scales and the RRC-ARM sub-scales of Family Attachment and Support and Social/Community Inclusion as family and social attachments have been shown to support wellbeing in adolescence (43). To correct for multiple comparisons, we applied a Bonferroni correction for 136 correlation tests with a corrected *p*-value threshold of 0.000368.

Cut-offs for COMPAS-W ‘flourishing’, ‘moderate’, and ‘languishing’ scores were determined once the COMPAS-W factor structure was confirmed and reliability analyses completed. These cut-offs were guided by matching *z*-scores (-1, 0, and +1) to the raw scores from the total adolescent group (clinical and non-clinical) and using these thresholds to define each category [a similar method was used and validated in adults (31)]. We then determined what percentage of participants from each of the clinical sub-samples fell in each ‘total sample’ cut-off category. A chi-square test was performed to determine whether there was a significant association between diagnosis and wellbeing cut-off group.

#### 2.4.2 Wellbeing by age, sex, and country of residence

Associations between wellbeing and key demographic variables were evaluated for age, sex (males vs females), and country of residence (Australia vs USA) in a multiple regression model. Age was included as a continuous and mean-centered variable. Based on previous wellbeing research in adolescents (42), we expected non-linear effects of age and therefore included age as a linear and quadratic term in the model.

#### 2.4.3 Wellbeing by diagnosis group

To investigate differences in wellbeing between the non-clinical and clinical groups (namely, developmental disorders, psychiatric disorders, or both developmental and psychiatric disorders), we conducted a Welch Two Sample t-test. Wellbeing scores were then compared across diagnoses with ANOVA analyses, followed by a Games-Howell *post hoc* test.

## 3 Results

### 3.1 Descriptive statistics

**Table 1** describes the characteristics of this adolescent sample (*n* = 1,078). Approximately half identified as male (51.67%). There was a fairly even spread of participants across each age group: 13 years (17.35%), 14 years (18.18%), 15 years (21.61%), 16 years (22.26%), and 17 years (20.59%). An approximately equal number of participants came from Australia (48.89%) and the United States of America (USA) (51.11%). A small percentage of participants reported being currently diagnosed with at least one developmental diagnosis (but not a psychiatric diagnosis) (8.35%), or at least one psychiatric diagnosis (but not a developmental diagnosis) (6.31%), or both types of diagnoses (at least one learning or developmental diagnosis and at least one psychiatric diagnosis) (6.22%), with the majority of participants reporting no current developmental or psychiatric diagnoses (79.13%).

**Table 1:** Descriptive statistics: number of participants by demographic variables (*n* = 1,078).

Demographic variables	% (*n*)
Sex	
Male	51.67 (557)
Female	48.33 (521)
Age (years)	
13	17.35 (187)
14	18.18 (196)
15	21.61 (233)
16	22.26 (240)
17	20.59 (222)
Country of residence	
Australia	48.89 (527)
USA	51.11 (551)
Diagnoses	
No clinical diagnosis	79.13 (853)
Learning or developmental disorder diagnosis only	8.35 (90)
Psychiatric or psychological diagnosis only	6.31 (68)
Both developmental and psychiatric diagnoses	6.22 (67)

### 3.2 Confirmatory factor analyses, reliability, and validity

Internal reliability using Cronbach alpha for the original COMPAS-W 26-item scale and sub-scales (*n* = 1,078) was as follows: COMPAS-W total scale (*α* = 0.896) and sub-scales (Composure, *α* = 0.735; Own-worth, *α* = 0.694; Mastery, *α* = 0.619; Positivity, *α* = 0.721; Achievement, *α* = 0.827; and Satisfaction, *α* = 0.839).

**Figure 1:**
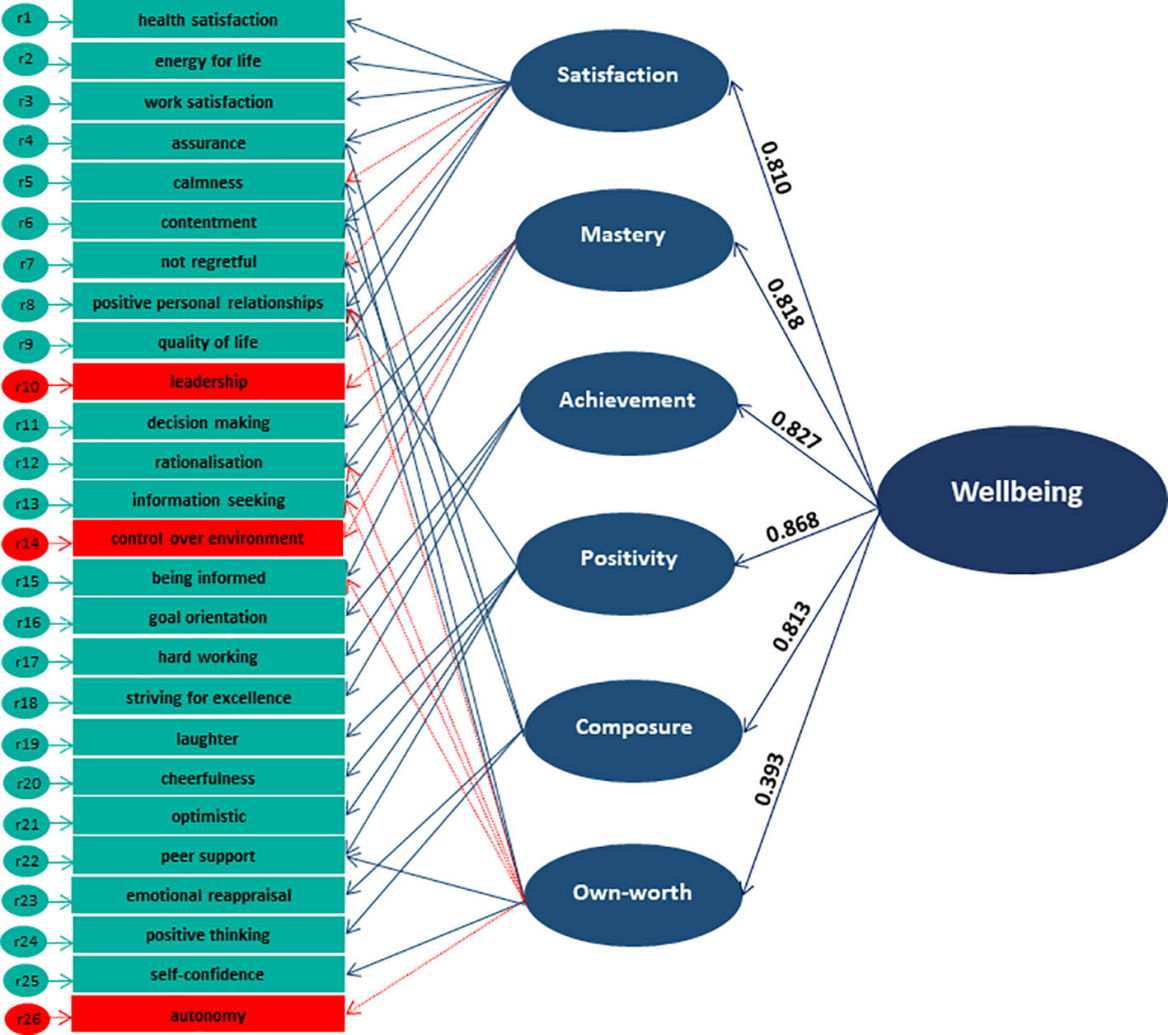
COMPAS-W Final Model (Model 4) derived from Second-Order Confirmatory Factor Analysis. Items 10, 14 and 26 (in red) were removed from the total scale for optimal fit in this adolescent sample. Items removed from the sub-scales are indicated by the dotted red lines. See **Table 2** for model estimates.

The second-order CFA on all 26 original items of the scale (Model 1), resulted in the following fit (χ*^2^* (283, 1,078) = 1941.680, *p* < 0.001, *CFI* = 0.863, *TLI* = 0.842, *RMSEA* = 0.072, *SRMR* = 0.074). In comparison to Model 1, three alternative nested models were then assessed. Model 2 tested the removal of item 26 from Own-worth and item 14 from Mastery; Model 3 tested the removal of item 26 from Own-worth and items 10 and 14 from Mastery; and Model 4 tested the removal of items 8, 12, 13, 15, and 26 from Own-worth, items 10 and 14 from Mastery, and items 5 and 7 from Satisfaction. All of these items were selected for removal because they either loaded non-significantly onto the subscale or had negative loadings. Model 4 demonstrated the best fit to the data (χ*^2^* (220, 1078) = 1439.395, *p* < 0.001, *CFI* = 0.893, *TLI* = 0.877, *RMSEA* = 0.070, *SRMR* = 0.095). Therefore, for Model 4, while items 8, 12, 13, 15, and 26 were removed from the Own-worth subscale, items 10 and 14 from Mastery, and items 5 and 7 from Satisfaction, due to cross-loadings of some of these items on other sub-scales, only items 10, 14 and 26 were removed from the entire scale (therefore, the total scale has 23 items instead of 26). See **Figure 1** for the final model structure, **Table 2** for the coefficients in the final model, **Table 3** for a comparison of all model fit indices, and **Supplementary Table 1** for information on the original 26-item COMPAS-W scale.

**Table 2:** COMPAS-W Second Order CFA Final Model (Model 4) Coefficients for **Figure 1**.

Scale	Item	Unstandardised Loadings	Standard Error	*p*-value	Standardised Loadings
*Composure*	4. assurance*	0.099	0.037	< 0.001	0.148
	6. contentment*	0.101	0.039	0.009	0.146
	23. emotional reappraisal	0.432	0.035	< 0.001	0.789
	24. positive thinking	0.404	0.032	< 0.001	0.772
*Own-worth*	5. calmness	0.900	0.056	< 0.001	0.779
	7. not regretful	0.864	0.046	< 0.001	0.728
	22. peer support*	0.067	0.025	0.008	0.088
	25. self-confidence	0.173	0.040	< 0.001	0.209
*Mastery*	11. decision making	0.289	0.027	< 0.001	0.563
	12. rationalisation	0.340	0.028	< 0.001	0.702
	13. information seeking	0.351	0.030	< 0.001	0.727
	15. being informed	0.337	0.029	< 0.001	0.665
*Positivity*	8. positive personal relationships*	0.159	0.028	< 0.001	0.357
	19. laughter	0.273	0.032	< 0.001	0.631
	20. cheerfulness	0.343	0.035	< 0.001	0.804
	21. optimistic	0.157	0.027	< 0.001	0.263
	22. peer support*	0.276	0.024	< 0.001	0.664
*Achievement*	16. goal orientation	0.396	0.026	< 0.001	0.774
	17. hard working	0.409	0.029	< 0.001	0.837
	18. striving for excellence	0.386	0.027	< 0.001	0.749
*Satisfaction*	1. health satisfaction	0.423	0.032	< 0.001	0.789
	2. energy for life	0.405	0.030	< 0.001	0.767
	3. work satisfaction	0.368	0.026	< 0.001	0.724
	4. assurance*	0.334	0.040	< 0.001	0.495
	6. contentment*	0.322	0.039	< 0.001	0.462
	8. positive personal relationships*	0.240	0.033	< 0.001	0.455
	9. quality of life	0.401	0.026	< 0.001	0.778
*TOTAL*	*Composure*	1.399	0.138	< 0.001	0.813
	*Own-worth*	0.427	0.071	< 0.001	0.393
	*Mastery*	1.422	0.156	< 0.001	0.818
	*Positivity*	1.752	0.220	< 0.001	0.868
	*Achievement*	1.472	0.131	< 0.001	0.827
	*Satisfaction*	1.380	0.140	< 0.001	0.810

Items with cross-loadings marked with an *.

**Table 3:** COMPAS-W second order CFA model fit statistics.

		*n*	chisq	*df*	*p*-value	CFI	TLI	RMSEA	SRMR
Model 1	26-items	1078	1941.680	283	<0.001	0.863	0.842	0.072	0.074
Model 2	Own-worth: Item 26 removed Mastery: Item 14 removed	1078	1585.707	236	<0.001	0.883	0.864	0.071	0.086
Model 3	Own-worth: Item 26 removed Mastery: Items 10 & 14 removed	1078	1407.620	214	<0.001	0.895	0.876	0.070	0.081
**Model 4**	**Own-worth: Items 8, 12, 13, 15, 26 removed;** **Mastery: Items 10 & 14 removed;** **Satisfaction: Items 5 & 7 removed**	**1078**	**1439.395**	**220**	**<0.001**	**0.893**	**0.877**	**0.070**	**0.095**

Best model (Model 4 in **bold)**. chisq, chi-square statistic; df, degrees of freedom; CFI, Comparative Fit Index; TLI, Tucker–Lewis index; RMSEA, Root Mean Square Error of Approximation; SRMR, Standardized Root Mean Square Residual.

Invariance testing for sex and country of residence revealed some differences. Testing for *configural invariance* showed that the same factor structure held for sex (χ*^2^* (440, 1,078) = 1740.259, *p* < 0.001, *CFI* = 0.881, *TLI* = 0.863, *RMSEA* = 0.074, *SRMR* = 0.093). However, chi-squared difference tests for *metric invariance* were significant (*Δ* χ*^2^ diff* = 71.194, *Δ df* = 26, and *p <* 0.001), indicating factor loadings were not equal across sex. Chi-squared difference test results for *scalar invariance* were also significant (*Δ* χ*^2^ diff* = 37.604, *Δ df* = 16, and *p <* 0.05) indicating both factor loadings and intercepts were not equal across sex (see **Table 4** for all chi-squared difference test fit statistics). When running the final model separately according to sex, for boys we found items 4 and 6 loaded insignificantly on Composure and item 22 loaded insignificantly on Own-worth, whereas for girls only item 22 loaded insignificantly on Own-worth. For country, testing for configural invariance showed that the same factor structure held across both Australian and American participants (χ*^2^* (440, 1,078) = 1744.192, *p* < 0.001, *CFI* = 0.880, *TLI* = 0.862, *RMSEA* = 0.074, *SRMR* = 0.090). However, chi-squared difference tests for metric invariance were significant (*Δ* χ*^2^ diff* = 54.239, *Δ df* = 26, and *p* < 0.001), indicating factor loadings were not equal across country; but chi-squared difference test results for scalar invariance were not significant (*Δ* χ*^2^ diff* = 19.105, *Δ df* = 16, and *p* > 0.05), meaning intercepts were equal across country. When we ran the final model for those from the USA only, we found items 4 and 6 loaded insignificantly on Composure, whereas for Australians, item 22 loaded insignificantly on Own-worth. Together, while the models demonstrated variance for specific COMPAS-W sub-scales, the total scale scoring for boys and girls, or Australians and Americans were unaffected, so total scores can still be used for comparison.

**Table 4:** Chi-squared difference test fit indices for assessing invariance across sex and country.

Sex								
Model	*df*	AIC	BIC	chisq	chisq diff	RMSEA	*df* diff	*p*-value
Model 1 (configural)	440	58172	58959	1740.3				
Model 2 (metric)	466	58191	58849	1811.5	71.194	0.057	26	< 0.001
Model 3 (scalar)	482	58197	58775	1849.1	37.604	0.050	16	0.002
Country								
Model	*df*	AIC	BIC	chisq	chisq diff	RMSEA	*df* diff	*p*-value
Model 1 (configural)	440	58158	58945	1744.2				
Model 2 (metric)	466	58160	58818	1798.4	54.239	0.045	26	< 0.001
Model 3 (scalar)	482	58147	58725	1817.5	19.105	0.019	16	0.263

The following results are all based on the final 23-item model. Internal reliability using Cronbach’s alpha for the new 23-item COMPAS-W model was again run for the total scale (*α* = 0.912) and sub-scales (Composure, *α* = 0.735; Own-worth, *α* = 0.601; Mastery, *α* = 0.757; Positivity, *α* = 0.721; Achievement, *α* = 0.827; and Satisfaction, *α* = 0.867), with demonstrated improvements in reliability compared to the original 26-item version for the total scale (previously 0.896), Mastery (previously 0.619), and Satisfaction (previously 0.839) scales. Reliability for Composure, Positivity, and Achievement remained the same, while Own-worth dropped slightly (from 0.694 to 0.601), most likely due to 5 items being removed from the subscale in the final model. Cronbach reliability results did suggest that the further removal of item 25 from the Own-worth sub-scale would have increased the Own-worth subscale reliability back up to 0.633, but we decided to keep this item in the model as it contributed significantly to the model and measured self-confidence which is important in Own-worth (and likely to become more important with age). In addition to Cronbach alpha reliability, we also calculated ‘composite reliability’ which takes into consideration factor loading weights of the CFA model (see **Supplementary Results**).

Correlations between the COMPAS-W sub-scales are presented in **Table 5**. Test-retest reliability over six weeks was good for the total scale (*r* = 0.845) and good for the sub-scales: Composure (*r* = 0.754), Own-worth (*r* = 0.743), Mastery (*r* = 0.715), Positivity (*r* = 0.750), Achievement (*r* = 0.750), and Satisfaction (*r* = 0.812).

**Table 5:** Inter-correlations of the COMPAS-W (23-item) total and sub-scales.

	COMPAS-W Total Scale	Composure	Own-worth	Mastery	Positivity	Achievement	Satisfaction
**COMPAS-W Total Scale**	––	< 0.001	< 0.001	< 0.001	< 0.001	< 0.001	< 0.001
**Composure**	.821^*^	––	< 0.001	< 0.001	< 0.001	< 0.001	< 0.001
**Own-worth**	.719^*^	.488^*^	––	< 0.001	< 0.001	< 0.001	< 0.001
**Mastery**	.722^*^	.517^*^	.337^*^	––	< 0.001	< 0.001	< 0.001
**Positivity**	.840^*^	.648^*^	.581^*^	.527^*^	––	< 0.001	< 0.001
**Achievement**	.749^*^	.537^*^	.368^*^	.647^*^	.552^*^	––	< 0.001
**Satisfaction**	.883^*^	.821^*^	.562^*^	.490^*^	.782^*^	.541^*^	––

Correlation values (r) presented below the diagonal, and significance (*p* values) presented above the diagonal.

Bonferroni-corrected *p*-value threshold for 21 tests is 0.00238. Tests which met the Bonferroni-corrected *p*-value threshold are indicated with an ^*^.

With regards to criterion validity, the scales which correlated the most positively with COMPAS-W were the RRC-ARM total scale (*r* = 0.660), RRC-ARM Social community inclusion/supports sub-scale (*r* = 0.640), PANAS positive affect sub-scale (*r* = 0.599), and the RRC-ARM Family attachments/support sub-scale (*r* = 0.553). The most negatively correlated scales with COMPAS-W were the SDQ total difficulties scale (*r* = -0.519), SDQ Hyperactivity sub-scale (*r* = -0.500), SDQ Emotional problems sub-scale (*r* = -0.462), DASS-21 scale (*r* = -0.462), Loneliness scale (*r* = -0.422), and the SDQ Peer problems score (*r* = -0.414) (see **Table 6**).

**Table 6:** Correlation Table of COMPAS-W (23-items) and Other Criterion Mental Health and Wellbeing Measures.

	COMPAS-W	DASS-21	EMO-SDQ	CON-SDQ	HYPER-SDQ	PEER-SDQ	PRO-SDQ	SDQ	RRC-ARM	RRC-SOC	RRC-FAM	LONE-LINESS	PANAS-P	PANAS-N	BULLY	BCS-ACT	BCS-DIS
**COMPAS-W**	–	< 0.001	< 0.001	< 0.001	< 0.001	< 0.001	< 0.001	< 0.001	< 0.001	< 0.001	< 0.001	< 0.001	< 0.001	< 0.001	< 0.001	< 0.001	< 0.001
**DASS-21**	-.462^*^	–	< 0.001	< 0.001	< 0.001	< 0.001	< 0.001	< 0.001	< 0.001	< 0.001	< 0.001	< 0.001	< 0.001	< 0.001	< 0.001	< 0.001	< 0.001
**EMO-SDQ**	-.462^*^	.734^*^	––	< 0.001	< 0.001	< 0.001	0.002	< 0.001	< 0.001	< 0.001	< 0.001	< 0.001	< 0.001	< 0.001	< 0.001	< 0.001	< 0.001
**CON-SDQ**	-.377^*^	.631^*^	.614^*^	––	< 0.001	< 0.001	< 0.001	< 0.001	< 0.001	< 0.001	< 0.001	< 0.001	0.083	< 0.001	< 0.001	< 0.001	< 0.001
**HYPER-SDQ**	-.500^*^	.557^*^	.578^*^	.565^*^	––	< 0.001	< 0.001	< 0.001	< 0.001	< 0.001	< 0.001	< 0.001	< 0.001	< 0.001	< 0.001	0.001	< 0.001
**PEER-SDQ**	-.414^*^	.546^*^	.603^*^	.537^*^	.441^*^	––	< 0.001	< 0.001	< 0.001	< 0.001	< 0.001	< 0.001	< 0.001	< 0.001	< 0.001	0.001	< 0.001
**PRO-SDQ**	.407^*^	-.161^*^	-.093	-.273^*^	-.322^*^	-.256^*^	––	< 0.001	< 0.001	< 0.001	< 0.001	< 0.001	< 0.001	< 0.001	< 0.001	0.001	0.339
**SDQ**	-.519^*^	.759^*^	0.882	.803^*^	.785^*^	.779^*^	-.262^*^	––	< 0.001	< 0.001	< 0.001	< 0.001	< 0.001	< 0.001	< 0.001	< 0.001	< 0.001
**RRC-ARM**	.660^*^	-.391^*^	-.349^*^	-.390^*^	-.459^*^	-.437^*^	.535^*^	-.476^*^	––	< 0.001	< 0.001	< 0.001	< 0.001	< 0.001	< 0.001	< 0.001	< 0.001
**RRC-SOC**	.640^*^	-.362^*^	-.332^*^	-.335^*^	-.412^*^	-.449^*^	.483^*^	-.445^*^	.938^*^	––	< 0.001	< 0.001	< 0.001	< 0.001	< 0.001	< 0.001	< 0.001
**RRC-FAM**	.553^*^	-.419^*^	-.375^*^	-.434^*^	-.426^*^	-.440^*^	.464^*^	-.492^*^	.903^*^	.789^*^	––	< 0.001	< 0.001	< 0.001	< 0.001	0.019	< 0.001
**LONELINESS**	-.422^*^	.585^*^	.563^*^	.539^*^	.456^*^	.518^*^	-.266^*^	.634^*^	-.494^*^	-.459^*^	-.521^*^	––	< 0.001	< 0.001	< 0.001	< 0.001	< 0.001
**PANAS-P**	.599^*^	-.129^*^	-.119^*^	-0.053	-.279^*^	-.111^*^	.384^*^	-.149^*^	.516^*^	.506^*^	.383^*^	-.211^*^	––	0.385	0.915	< 0.001	0.006
**PANAS-N**	-.396^*^	.699^*^	.706^*^	.579^*^	.482^*^	.541^*^	-.121^*^	.716^*^	-.293^*^	-.269^*^	-.353^*^	.543^*^	0.026	––	< 0.001	< 0.001	< 0.001
**BULLY**	-.271^*^	.607^*^	.543^*^	.549^*^	.394^*^	.506^*^	-.111^*^	.610^*^	-.262^*^	-.235^*^	-.312^*^	.494^*^	0.003	.559^*^	––	< 0.001	< 0.001
**BCS-ACT**	.109^*^	.367^*^	.292^*^	.287^*^	0.101	0.221	0.208	.288^*^	.182^*^	.178^*^	0.071	0.179	.311^*^	.343^*^	.352^*^	––	< 0.001
**BCS-DIS**	-.222^*^	.677^*^	.635^*^	.586^*^	.400^*^	0.511	-0.029	.663^*^	-.188^*^	-.161^*^	-.277^*^	.533^*^	0.084	.653^*^	.605^*^	.706^*^	––

Correlation values (*r*) presented below the diagonal, and *p* values presented above the diagonal. DASS-21, Depression Anxiety Stress Scale (log-transformed); SDQ, SDQ; EMO-SDQ, SDQ Emotional problems score; CON-SDQ, SDQ Conduct problems score (log-transformed); HYPER-SDQ, SDQ Hyperactivity score; PEER-SDQ, SDQ Peer problems score; PRO-SDQ, SDQ Prosocial score; RRC-ARM, The Resilience Research Centre Adult Resilience Measure; RRC-SOC, RRC-ARM Social sub-scale; RRC-FAM, RRC-ARM Family sub-scale; LONELINESS, Loneliness scale; PANAS-P, PANAS Positive Affect sub-scale; PANAS-N, PANAS Negative Affect sub-scale (log-transformed); BULLY, cyberbullying (log-transformed); BCS-ACT, BCS active coping sub-scale; BCS-DIS, BCS disengaged coping sub-scale (log-transformed); Bonferroni-corrected *p*-value threshold for 136 tests is 0.000368.

*Met Bonferroni-corrected *p*-value threshold for 136 tests.

### 3.3 Wellbeing by age, sex, and country of residence

**Figure 2:**
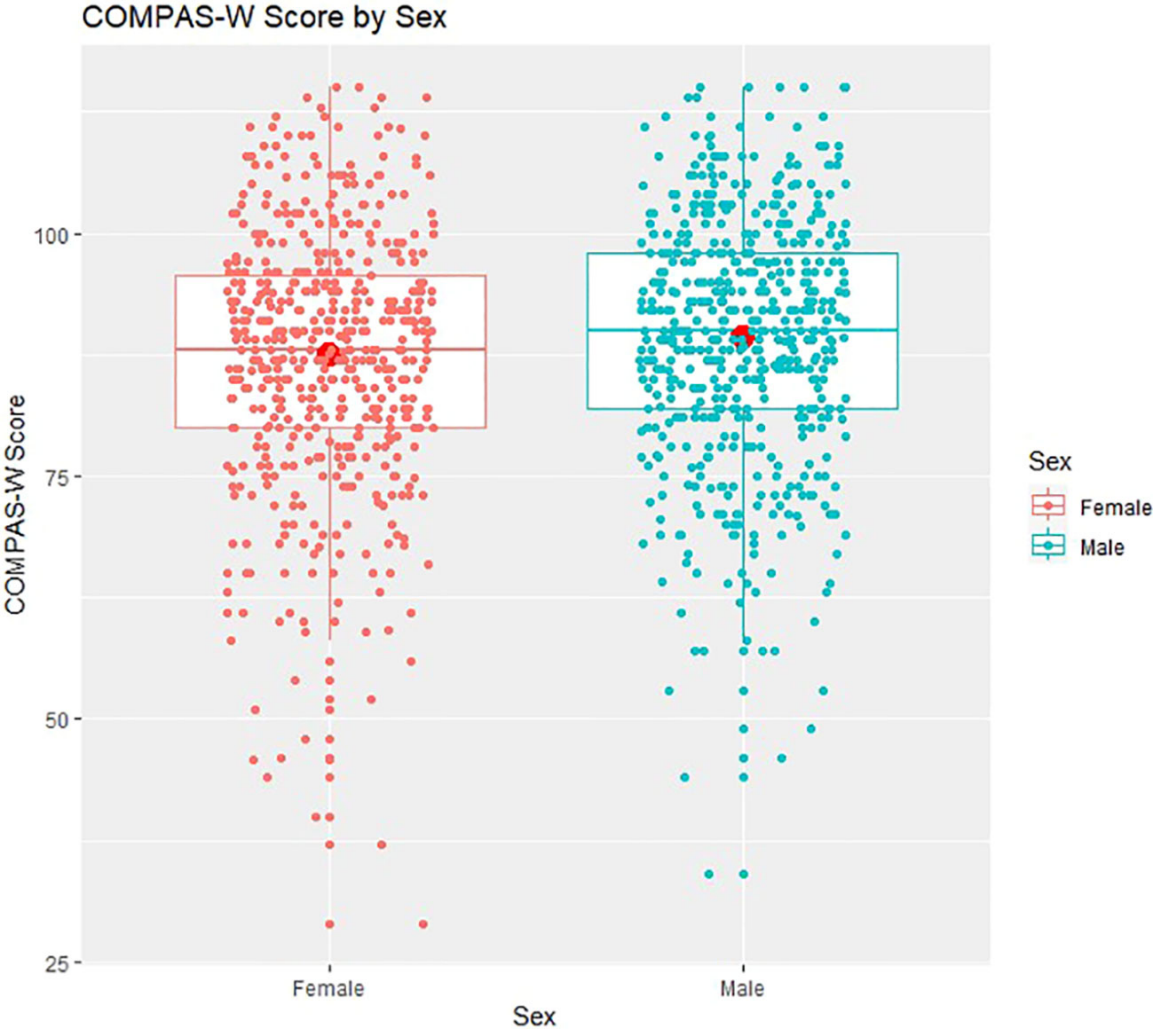
COMPAS-W wellbeing scores by sex. Boxplots of COMPAS-W (23 items) scores by sex showing means (large centre dots), medians (mid-lines), interquartile ranges, minimum and maximum values; overlayed with corresponding distribution of group values. On average, compared to females, males scored significantly higher in wellbeing.

**Figure 3:**
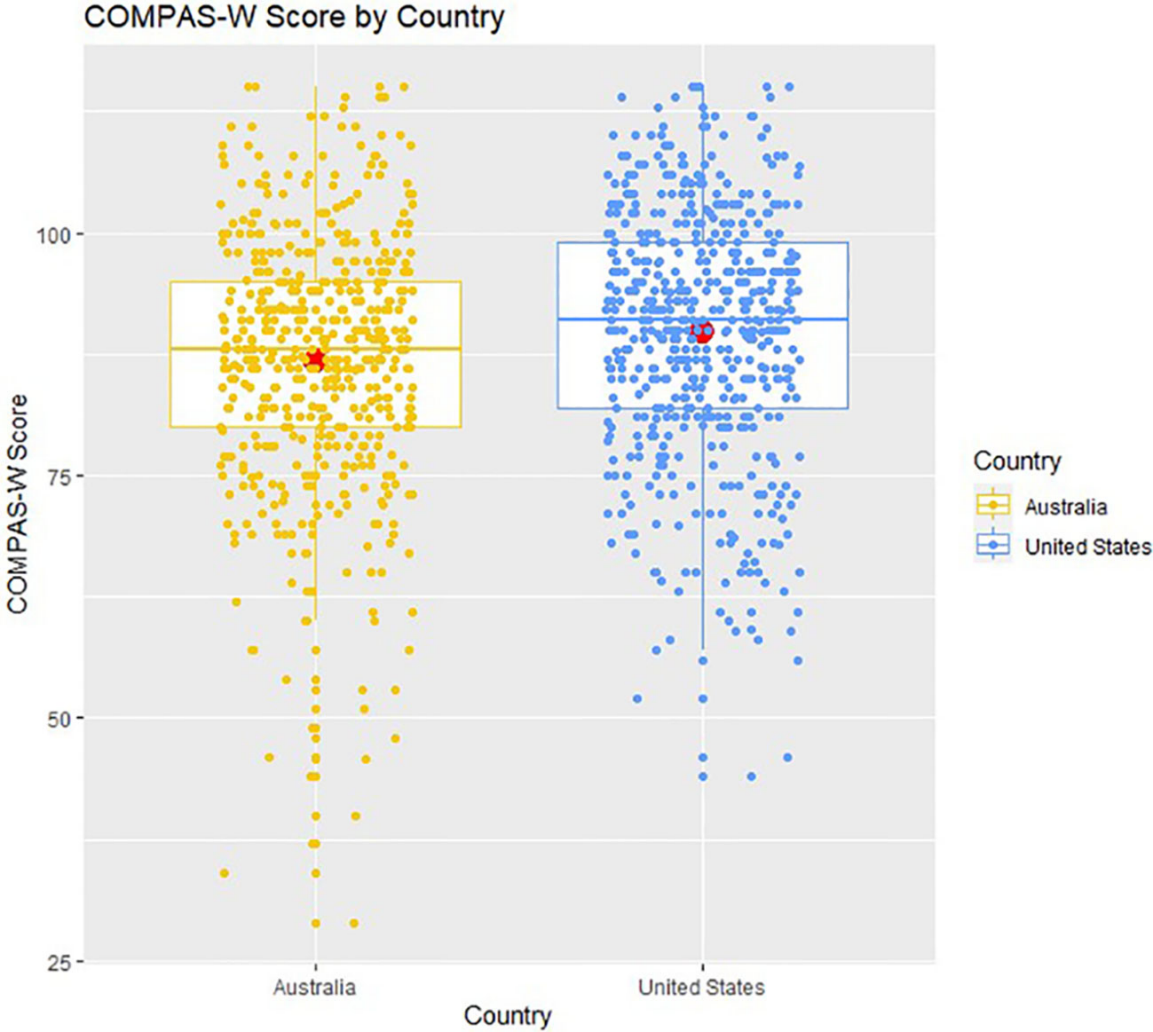
COMPAS-W wellbeing scores by country. Boxplots of COMPAS-W (23 items) scores by country showing means (large centre dots), medians (mid-lines), interquartile ranges, minimum and maximum values; overlayed with corresponding distribution of group values. On average, compared to Australians, Americans scored significantly higher in wellbeing.

The multiple regression results showed that age was not a significant predictor of wellbeing (either linearly or quadratically). However, on average, males scored 1.731 points significantly higher in wellbeing (*95% CI* [0.190, 3.272], *p* = 0.028) compared with females, controlling for age and country. In addition, compared with Australians, Americans scored 3.042 points significantly higher in wellbeing (*95% CI* [1.502, 4.582], *p* < 0.001) on average, controlling for age and sex. Mean wellbeing scores for sex and country are presented in **Figures 2**, **3**.

### 3.4 Wellbeing by diagnosis group

**Figure 4:**
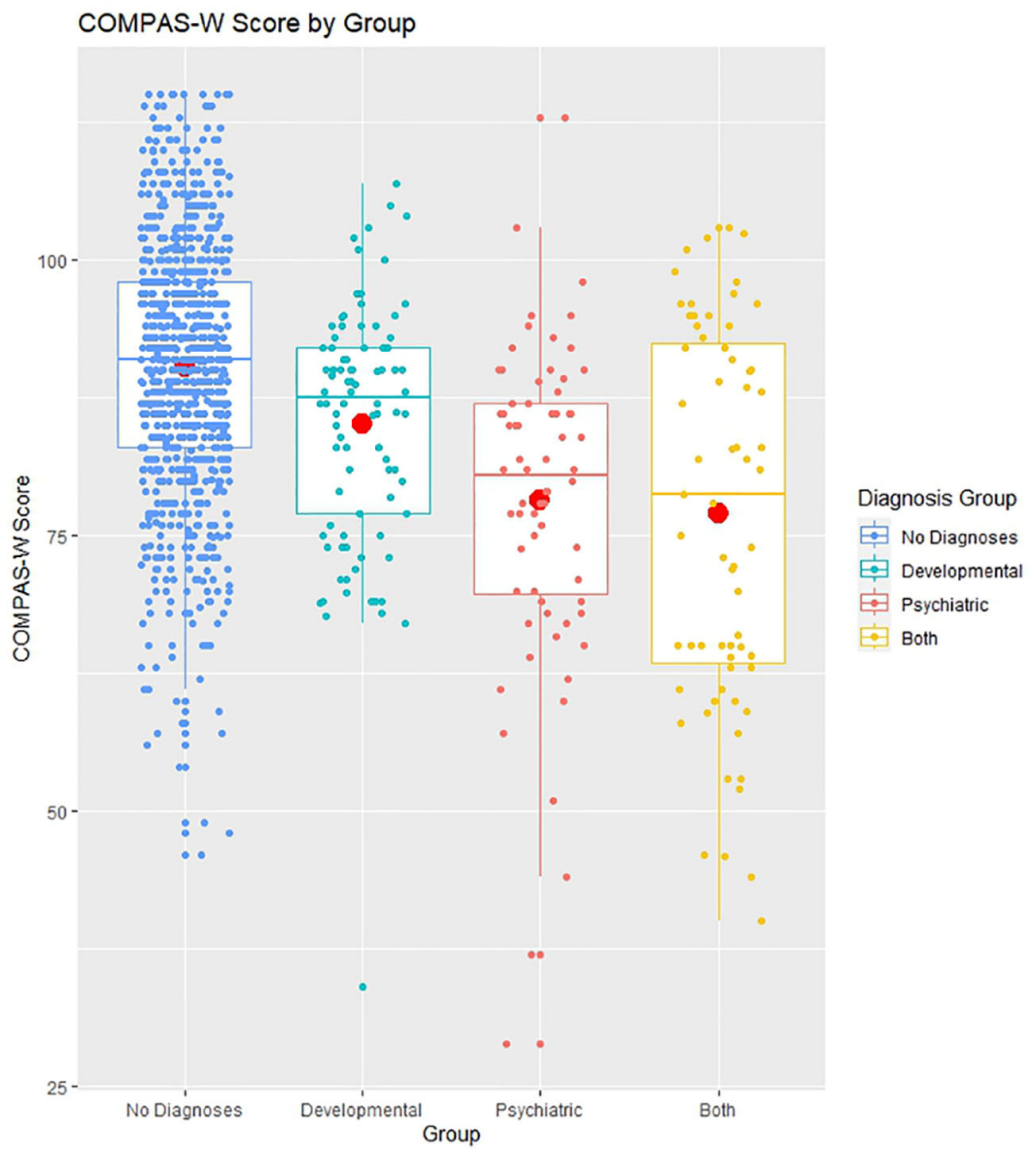
COMPAS-W wellbeing scores by diagnosis group. Boxplots of COMPAS-W (23 items) scores by diagnosis group, showing means (large centre dots), medians (mid-lines), interquartile ranges, minimum and maximum values; overlayed with corresponding distribution of group values. The means of the developmental diagnosis group, psychiatric diagnosis group, and both developmental and psychiatric diagnoses group all differed significantly to the mean of the no diagnoses (non-clinical) group.

**Figure 5:**
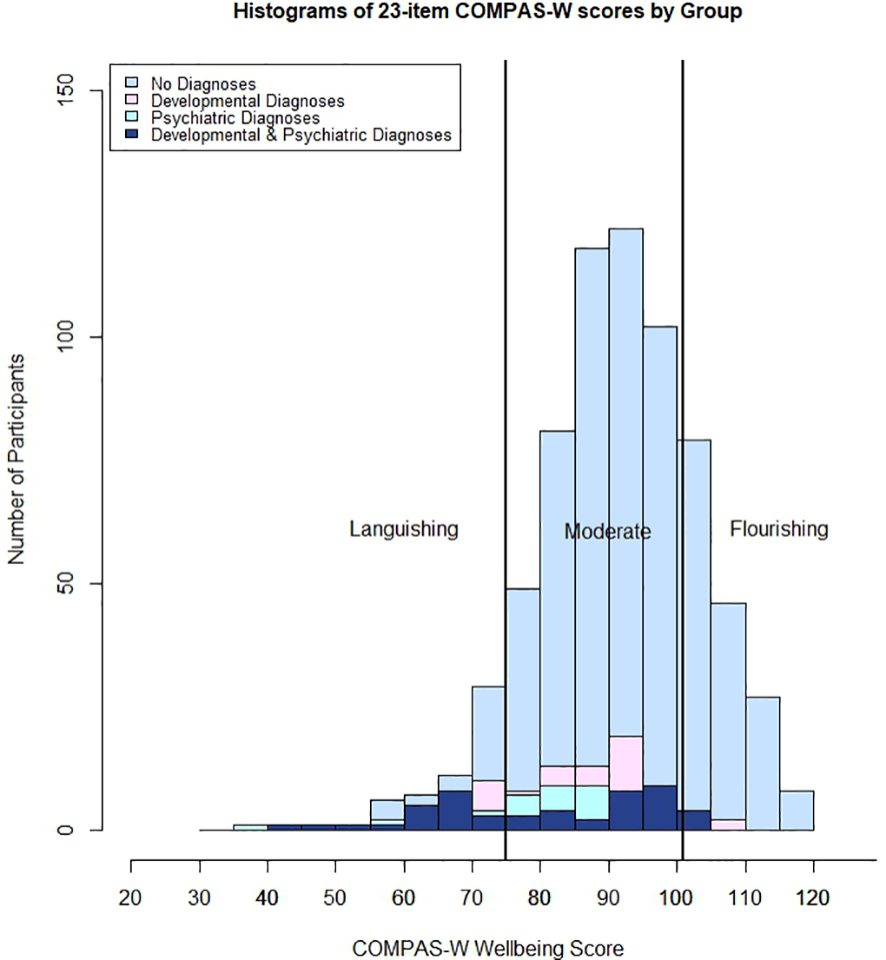
Histogram of COMPAS-W (23 items) scores by diagnosis group. No Diagnoses (non-clinical) = no developmental or psychiatric diagnoses reported; Developmental Diagnoses = at least one developmental diagnosis reported; Psychiatric Diagnoses = at least one psychiatric diagnosis reported; and Both Diagnoses = at least one developmental and one psychiatric diagnosis reported.

Mean wellbeing scores by diagnosis group are presented in **Figure 4**, and the distribution of wellbeing category scores for the four diagnosis groups are contrasted in **Figure 5**.

For mean wellbeing scores, the *p*-value from Bartlett’s test was less than 0.05, therefore we could reject the null hypothesis that each of the four diagnoses groups (non-clinical, developmental diagnoses, psychiatric diagnosis, and both developmental and psychiatric diagnoses) have the same variance, so we proceeded to perform Welch’s ANOVA for unequal variances. The overall *p*-value from the ANOVA was less than 0.05, therefore we rejected the null hypothesis that wellbeing scores were equal between the four diagnosis groups. *Post-hoc* tests showed that, compared with non-clinical participants (*M* = 90.375, *SE* = 0.400), those with diagnoses reported significantly lower mean wellbeing on average (developmental diagnoses: *M* = 85.088, *SE* = 1.188, *p* < 0.001, psychiatric diagnoses: *M* = 78.189, *SE* = 1.758, *p* < 0.001, developmental and psychiatric diagnoses: *M* = 77.079, *SE* = 2.116, *p* < 0.001). Those with only a psychiatric diagnosis had significantly lower mean wellbeing compared with those with only a developmental diagnosis (*p* = 0.008) and those with both developmental and psychiatric diagnoses also had lower mean wellbeing compared with those with only a developmental diagnosis (*p* = 0.007). However, there was no difference in average wellbeing between those with both developmental and psychiatric diagnoses compared with those with only psychiatric diagnoses (*p* = 0.978) (see **Figure 4**).

When instead considering wellbeing categories (rather than means), the prevalence of a flourishing, moderate, or languishing state of wellbeing differed between groups with and without a developmental or psychiatric diagnosis. There was a significant association between diagnosis group and wellbeing category, (χ*^2^* (6) = 100.548, *p* < 0.001)(see **Figure 5**). For prevalence estimates, please refer to **Table 7**. **Supplementary Table 2** presents the comparative raw scores for the COMPAS-W (26 items) and COMPAS-W (23 items), for both the total scale and sub-scales. Corresponding cut-off scores for flourishing, moderate, and languishing groups for the 23-item COMPAS-W scale are presented for the whole sample and non-clinical (healthy) participants in **Supplementary Table 3**.

**Table 7:** COMPAS-W (23 item) scale cut-offs: percentage of participants in each range by diagnosis group.

Whole Sample*	Non-clinical participants (no diagnoses)	Developmental diagnosis	Psychiatric diagnosis	Developmental & Psychiatric diagnoses
Languishing ≤ 75	10.67%	23.33%	33.82%	47.76%
Moderate 76–100	71.70%	71.07%	64.68%	46.24%
Flourishing ≥ 101	17.58%	5.56%	1.47%	5.97%

*Whole sample includes clinical and non-clinical participants. For corresponding category cut-offs based on the non-clinical (healthy) sample alone, refer to **Supplementary Table 3**.

Chi-square analyses showed there was a significant association between diagnosis group and wellbeing state, χ^2^ (6) = 100.548, *p* < 0.001.

**Supplementary Table 2** presents the comparative raw scores for the COMPAS-W (26 items) and COMPAS-W (23 items), both total scale and sub-scales. Corresponding cut-offs for languishing, moderate and flourishing groups for the 23-item COMPAS-W scale are presented for the whole sample and non-clinical (healthy) participants in **Supplementary Table 3**.

## 4 Discussion

### 4.1 Summary of outcomes

Adolescence presents a period of risk for mental ill-health, but also a window of opportunity towards a state of flourishing. Yet no comprehensive mental wellbeing measures have been thoroughly validated exclusively for adolescents thus far. Therefore, our aims were to confirm the factor structure and establish internal and test-retest reliability and criterion validity of COMPAS-W in an adolescent sample (13–17 years); to examine whether mental wellbeing in adolescence differed by key demographic factors such as age, sex, and country; and to determine the degree to which mental wellbeing differs in adolescents who have a developmental or/and psychiatric diagnosis compared to those who are non-clinical.

We found the best fitting CFA model for COMPAS-W in this adolescent sample was a second-order factor model that comprised a total scale of 23 items (i.e., dropping 3 items from the original scale) and six sub-scales. The model fit was good with final indices reported (*CFI* = 0.893, *TLI* = 0.877, *RMSEA* = 0.070, *SRMR* = 0.095). While there is potential scope for further model improvement based on rule of thumb upper metrics (i.e., *CFI* ≥ 0.95, *TLI* ≥ 0.95, *RMSEA* ≤ 0.08, *SRMR* ≤ 0.08 (52)), it is possible our slightly lower fit indices are due to the use of the second-order factor model which tends to be more restricted (but parsimonious) relative to other less restricted (but more complex) CFA models (e.g., bi-factor model, which may result in better fit statistics (55), We chose the second-order factor model as its hierarchical structure aligned better with the way the original COMPAS-W sub-scales were designed to load on to one common factor, and this resulted in a satisfactory fit. Indeed, overall internal reliability for the new COMPAS-W 23-item scale (*α* = 0.912) was improved from the original 26-item version in the current sample (*α* = 0.896), and compared to the 26-item version in a comparative international sample of 194 adolescents (*α* = 0.883) (32) which supports the credibility of this revision for adolescent samples. The smaller international sample included slightly younger participants (12–17 years, *M* = 13.9, *SD* = 1.36) recruited from a broader range of countries (Australia, Canada, China, and New Zealand) which may also account for slightly different results (albeit, this previous sample comprised a similar sex distribution of 52% males, 46% females, and 2% sex undisclosed).

The COMPAS-W also proved to be a valid measure of wellbeing in adolescents for a range of other tests. For instance, good criterion validity was demonstrated by strong positive correlations with related measures assessing resiliency resources such as the RRC-ARM, and moderate negative correlations with related measures on the mental ill-health continuum such as the SDQ and DASS-21, as expected. The COMPAS-W also performed well when comparing its reliability results to those of other scales. The COMPAS-W has a higher total scale internal reliability (*α* = 0.912) compared to the Stirling Children’s Well-being Scale (*α* = 0.847) (27) and Warwick-Edinburgh Mental Well-being Scale (*α* = 0.870) (23). In addition, aside from the School Wellbeing Scale, the COMPAS-W is the only scale validated for adolescents that provides insight into a young person’s wellbeing beyond their total score and into their wellbeing subcomponents, thereby indicating which areas of wellbeing a person may require more support in. In comparison, the School Wellbeing Scale measures four subfactors (interpersonal wellbeing, life satisfaction, competence, and negative emotion), however internal reliabilities for these subfactors have not yet been reported. In contrast, the COMPAS-W is able to inform us of an adolescent’s wellbeing across six different aspects with good internal reliability: Composure (*a*= 0.735), Own-worth (*a* = 0.601), Mastery (*a* = 0.757), Positivity (*a* = 0.721), Achievement (*a* = 0.827), and Satisfaction (*a* = 0.867), in addition to their overall wellbeing score. Further, the COMPAS-W total scale has higher test-retest reliability (*r* = 0.845) compared with the Stirling Children’s Well-being Scale (*r* = 0.752) (27) despite the COMPAS-W retest period being longer (six weeks) than the Stirling Children’s Well-being Scale (one week), suggesting the COMPAS-W performs more consistently over time.

With regards to differences in wellbeing across demographic characteristics such as age, no variation was found. Similar total scores were observed between younger and older adolescents within the sample, however the narrow age bracket (13–17 years) may have accounted for a lack of significant differences in wellbeing observed by age. Other wellbeing studies with similar age ranges (13–16 years) also failed to find any variation in wellbeing scores with age for the Warwick-Edinburgh Mental Well-being Scale (23) and Short Warwick-Edinburgh Mental Well-being Scale (26). Validation studies in psychological distress also reported no significant differences in DASS-21 scores across those aged 11–15 years (56) or 13–18 years (57). In contrast, Keyes’s study of 12–18-year-old young people reported a small negative correlation between age and mental health (*r*  = −.07; *p* < 0.02) (11) and the prevalence of flourishing also dropped from younger (12–14 years) to older adolescents (15–18 years) (4). Renshaw and Bolognino (42) also found medium-sized differences in subjective wellbeing between younger (10–11 years) and older children (14–15 years and 15–16 years), but small differences in psychological distress between younger (10–11 years) and older children (14–15 years and 15–16 years). Both of these studies may have observed differences in wellbeing by age given they included young people during early adolescent periods, thus capturing an earlier age of onset for various conditions. For instance, the median age of onset for attention-deficit/hyperactivity disorder, phobias, and separation anxiety disorder may occur as early as 7 years, while the age of onset for other anxiety disorders ranges from 7–14 years (58). Therefore, in future studies it would be helpful to include young children through to young adults to see if any variability in wellbeing or distress can be observed by age, as well as stratification by specific diagnoses.

In terms of sex differences, males reported slightly higher wellbeing on average than females. Although this difference between the sexes was significant, the effect was quite small. Other wellbeing studies have also found small differences in wellbeing by sex. Tennant’s (22) Warwick-Edinburgh Mental Well-being Scale study of participants 16 to 75 years and older reported a difference of one point in wellbeing scores between males and females. In a similar age range (13–16 years) to our sample, Clarke (23) also found Warwick-Edinburgh Mental Well-being Scale scores to be slightly higher in males compared with females, however this effect disappeared after adjusting for age. Adolescent studies in psychological distress have shown a similar pattern but in the opposing direction whereby girls reported significantly higher depression and anxiety scores than boys (56, 57). These sex differences suggest biological differences may be contributing to the discrepancy in psychological distress between the sexes. For instance, research has suggested that hormones may have an effect on female mental health. Oestradiol and progesterone have been hypothesised to make females more vulnerable to the onset of anxiety diagnoses, but also potentially support the maintenance of existing anxiety disorders (59). Findings to date also indicate that females with an anxiety diagnosis may be at greater risk of experiencing anxiety during the premenstrual phase of their cycle (60). Therefore, in future research, it would be helpful to investigate how wellbeing can support those with clinical diagnoses at different points of their cycle. In addition, when examining various aspects of wellbeing, researchers need to consider sex differences. When comparing the fit of the COMPAS-W model structure for sex in terms of configural invariance, results showed that the factors and pattern of their loadings were the same across sex; however, for metric invariance, we found significant differences suggesting that the factor loadings were not equal between boys and girls. We also found significant effects for our scalar invariance model, therefore both factor loadings and intercepts were not equal between the sexes. This may be simply a characteristic of the current sample. Similarly, Hunter et al’s testing of the short Warwick-Edinburgh Mental Well-being Scale found a violation of invariance in their scalar model (26). For the COMPAS-W, this applied to sub-scales only (not total scores). In any case, in future studies using COMPAS-W to compare boys to girls, total scores and subscale scores can still be scored in the same way as proposed here in the 23-item version, but there may be differences in estimate loadings between boys and girls.

With regards to country of residence, Australians reported slightly lower wellbeing than American participants. Other studies have also suggested variations in wellbeing between different countries although the evidence is equivocal. For example, Cosma et al’s multi-level analyses of life satisfaction in 11, 13, and 15 year old young people across 36 countries showed an increase in model fit when country level was added to their model, thus indicating significant unexplained variance in life satisfaction across countries (61); however, the authors did not directly compare mean differences in life satisfaction between the individual countries. Marquez’s study also reported differences in life satisfaction over time between the sexes for 15-year olds living in Scotland, England, Wales, Northern Ireland, the United States, Japan, Ireland, and France, but did not compare life satisfaction between countries (46). Therefore, further research is needed on country differences in wellbeing and country-specific influences on adolescent wellbeing. One possible explanation for the difference in wellbeing between countries in our study is that our data was collected during a period when Australia and the USA experienced differing prevalences of COVID-19. In June 2020, Australia experienced one of two peaks in COVID-19 cases and deaths for that year (62) whereas in the USA, the number of new cases per day were at a minimum (63). This may explain why our results showed that Americans reported slightly higher average wellbeing than Australians, after controlling for age and sex during data collection. Further, researchers may need to consider country differences when assessing different aspects of wellbeing. When comparing the fit of the COMPAS-W model for country, configural invariance testing provided support for the same factor structure across both countries, however for metric invariance, we found significant model differences suggesting that the factor loadings were not equal between Australian and American participants. However, we did not find significant results in our scalar invariance test, therefore intercepts may be equivalent between the countries. Again, for the COMPAS-W, this applied to 3 items in the sub-scales only (not total scores).

Our third aim was to examine wellbeing by diagnosis group. There was a clear difference in wellbeing category scores between non-clinical participants and those with diagnoses (developmental, psychiatric, and those with both developmental and psychiatric diagnoses). For instance, in non-clinical (‘healthy’) adolescents, 10.67% reported languishing wellbeing, relative to 71.70% and 17.58% reporting moderate and flourishing wellbeing, respectively. In comparison, all clinical groups also demonstrated varying levels of high and low wellbeing. For example, while 33.82% of participants with a psychiatric diagnosis reported languishing levels of wellbeing, 64.68% reported moderate wellbeing, and 1.47% reported flourishing states. Of participants with a developmental diagnosis, 23.33% were languishing, while 71.07% reported moderate wellbeing, and 5.56% were flourishing. For the group with both a psychiatric and a developmental diagnosis, 47.76% reported languishing, 46.24% a state of moderate wellbeing, and 5.97% a state of flourishing. Further, chi-square results showed a significant association between diagnosis group and state of wellbeing. These results align with the dual-continua model of mental health validated by Keyes (47) and Greenspoon and Saklofske (48) which suggests that psychopathology and mental wellbeing exist on separate continua rather than on the same common dimension. Thus, even though mental wellbeing and mental ill health are negatively correlated overall, when we observe the varying prevalence of low, moderate, and high wellbeing between the diagnosis groups, we can observe the nuances in prevalence. It is therefore possible to experience contrasting levels of psychopathology and wellbeing concurrently, even in individuals with clinical diagnoses, depending on the state of their condition and how it is managed.

### 4.2 Strengths and limitations

There are several strengths to this study. First, COMPAS-W, a comprehensive measure of wellbeing including measures of both subjective and psychological wellbeing, was validated for adolescents. While the COMPAS-W was not specifically designed for adolescents but for adults, its’ reliability and validity were established in the current adolescent sample, and replicating results we previously demonstrated in another independent adolescent sample of 12–17 year olds (32). In addition, validity of the scale was established in the current sample using the adolescent SDQ scale as a criterion measure, which showed substantial correlations with the COMPAS-W for the total SDQ score (*r* = -.519) and SDQ sub-scales (*r* ranging from -.377 to -.500). In new work, we are currently validating an adapted version of COMPAS-W suitable specifically for younger children aged 5 to 12 years called COMPAS-KIDS, which could be used in younger age groups (Lam et al., in prep). In comparison, most other existing wellbeing scales for adolescents only measure subjective wellbeing or sparsely cover both subjective and psychological wellbeing dimensions. Another study strength is that this is the first wellbeing validation study specifically targeting adolescence, a critical period of brain development during which core neural networks are impacted (64). In contrast, most previous wellbeing studies focused on averaged results across age whereby adolescents are included within a larger sample of children or adults. However, as childhood and adulthood are very different stages to adolescence, wellbeing outcomes that result from a mix of age groups may not truly represent the validity of measures for adolescents. Our study also examined wellbeing by diagnoses groups, supporting a dual-continua model of mental health in adolescents which has only been recognised by two other studies (42, 47) discussed above. We also thoroughly assessed the psychometric properties of the COMPAS-W for adolescents including CFA, measurement invariance testing for sex and country, validity, and reliability analyses whereas previous validation studies in adolescents have only focussed on factor analyses or validity and reliability. Our analyses were performed with a large sample, with fairly even age, sex, and country distributions, thus enabling a statistical comparison of results. This study’s sample was recruited from a broad population base, in comparison to many studies which only recruit adolescents from schools (12, 13, 23, 26–28). Recruiting exclusively from schools may generate bias or restrict the external validity of study results if for example, all the students in a school were of a particular socio-economic or cultural background, or influenced by the same school culture or wellbeing interventions.

Despite these strengths, there are several limitations also worth noting. First, as this study was conducted during the COVID-19 pandemic (with baseline data collected from May 1^st^ to June 10^th^, 2020) it is plausible that COVID-19 may have influenced country effects, diagnosis prevalence, or wellbeing levels in our sample. In addition, COVID-19 may have influenced the prevalence of psychiatric diagnoses in the data collection period. During April-September 2020, Straub et al. (65) reported in a large American sample, that the prevalence of anxiety, ADHD, and eating disorders (but not depression) increased in girls aged 13–18 years at a faster rate during the pandemic (compared to pre-pandemic rates). Similarly, in an analysis of Australian national data comparing pre-pandemic to pandemic periods, Khan et al. found a statistically significant increase in monthly average inpatient admissions and emergency department attendances in children across all mental health conditions, with the highest increase found in those 12–14 years and 15–18 years (66). Therefore, it would be interesting to compare our study results with those at a post-pandemic timepoint to see if wellbeing and prevalence of diagnoses has changed and what impact this may have on related outcomes. Another limitation is that psychiatric diagnosis was self-reported and not assessed using clinical interview, so while this is a common approach to measuring illness, this group may have been under or over represented in our results. The other limitation worth noting is the slightly lower reliability estimate for the Own-worth scale (Cronbach’s alpha for the 26-item scale, *α* = 0.694 dropped to 0.601 for the 23-item scale) which is likely due to multiple items being removed from the Own-worth subscale in the final model. Cronbach reliability results did suggest that the further removal of item 25 from the Own-worth subscale would have increased the Own-worth subscale reliability back up to 0.633, but we decided to keep this item in the model as it contributed significantly to the model and measured self-confidence which is important in Own-worth (and likely to become more important with age). Notably, in our previous adult sample (31), the items loaded more strongly onto the Own-worth subscale than in the current adolescent sample, and the Own-worth subscale was more reliable than some of the other sub-scales in the adult sample. This demonstrates that the Own-worth scale may contain items that load lower in younger samples but that become stronger with age and development. To put these results in context, in comparison to other wellbeing scales used in adolescents, the COMPAS-W performs quite well – as mentioned earlier, no other study has yet provided insight into a young person’s wellbeing beyond their total score. For instance, McLellan and Steward’s School Wellbeing Scale (28) measures four subfactors of wellbeing, yet internal reliabilities have not yet been reported. So, the outcomes from this study are a first that need to be replicated for other samples and scales. Finally, our results showed no variation in wellbeing by age. While this is not necessarily a limitation, future analyses should include younger children through to young adults (to capture earlier and later onset of various diagnoses) to see if variability in wellbeing can be observed by age.

### 4.3 Conclusion

Overall, the 23-item COMPAS-W Wellbeing Scale is a useful measure of subjective and psychological wellbeing for adolescents aged 13–17 years. Although there is room for improvement in measuring an adolescent’s Own-worth (which may improve with development and age as seen in our adult sample), results indicate the COMPAS-W provides reliable insight into several key subcomponents of wellbeing in addition to overall wellbeing. The COMPAS-W can be used to support a dual-continua model of mental health showing that even if adolescents experience psychopathology or developmental conditions, they can still experience moderate to high wellbeing. The 23-item version of COMPAS-W may be useful as a clinical measure to assess and support adolescents’ mental health, and as a research tool to further investigate underlying mechanisms of wellbeing and resilience in young people, with the 26-item version (31) still relevant to adult samples. Given the COMPAS-W is also used to measure wellbeing in adults, we will have the means to observe the transition in wellbeing from adolescence to adulthood using these tools.

## Data availability statement

The datasets presented in this article are not readily available because of ethical requirements. Requests to access the datasets should be directed to JG, j.gatt@unsw.edu.au.

## Ethics statement

The study was approved by the Human Research Ethics Committee of the University of New South Wales (UNSW), Sydney, Australia (HC200150). The studies were conducted in accordance with the local legislation and institutional requirements. Written informed consent was required from participants over the age of 18 years. For those participants under the age of 18 years, written informed consent was initially required from the participant's parent/legal guardian, followed by the participant's implied consent on the participant's completion of the survey.

## Author contributions

JL: Writing – review & editing, Writing – original draft, Validation, Investigation, Funding acquisition, Formal analysis. HP: Writing – review & editing, Resources, Project administration, Funding acquisition, Data curation. JG: Writing – review & editing, Supervision, Resources, Methodology, Funding acquisition, Conceptualization.
