# Correction: Measuring mental wellbeing in clinical and non-clinical adolescents using the COMPAS-W Wellbeing Scale

**DOI:** 10.3389/fpsyt.2026.1876633

**Published:** 2026-05-27

**Authors:** Janine R. Lam, Haeme R. P. Park, Justine M. Gatt

**Affiliations:** 1 Centre for Wellbeing, Resilience and Recovery, Neuroscience Research Australia, Sydney, NSW, Australia; 2 School of Psychology, University of New South Wales, Sydney, NSW, Australia

**Keywords:** mental health, well-being, adolescence, psychiatric disorders, developmental disorders, psychometric testing, reliability, validity

The **Supplementary material** contained the original 26-item COMPAS-W scale validated in adults and tested in the current adolescent sample. This proprietary instrument was not intended for unrestricted use and has been removed from the **Supplementary material** with directions to contact the corresponding author for further inquiries.

A correction has been made to **3 Results**, *3.2 Confirmatory factor analyses, reliability, and validity*, Paragraph 2. The sentence originally stated “See **Figure 1** for the final model structure, **Table 2** for the coefficients in the final model, **Table 3** for a comparison of all model fit indices, and **Supplementary Table 1** for 26 original items.” The corrected sentence states “See **Figure 1** for the final model structure, **Table 2** for the coefficients in the final model, **Table 3** for a comparison of all model fit indices, and **Supplementary Table 1** for information on the original 26-item COMPAS-W scale.”

Since original publication, the intellectual property rights to the COMPAS-W scale have been assigned to Tilt Your Life Pty Ltd, of which Dr. Justine Gatt is Founder and Director. The **Conflict of Interest** statement has therefore been updated to read:

“The intellectual property rights to the COMPAS-W scale are assigned to Tilt Your Life Pty Ltd, of which JG is Founder and Director. The scale is available for non-commercial academic and research use and is licensed for commercial use via Tilt Your Life Pty Ltd (contact: justine.gatt@gmail.com).

The remaining author(s) declare that the research was conducted in the absence of any commercial or financial relationships that could be construed as a potential conflict of interest.”

The original published findings are unaffected by this amendment.

The original version of this article has been updated.

