# Correction: Measuring mental wellbeing in clinical and non-clinical adolescents using the COMPAS-W Wellbeing Scale

**DOI:** 10.3389/fpsyt.2026.1907005

**Published:** 2026-07-27

**Authors:** Janine R. Lam, Haeme R. P. Park, Justine M. Gatt

**Affiliations:** 1 Centre for Wellbeing, Resilience and Recovery, Neuroscience Research Australia, Sydney, NSW, Australia; 2 School of Psychology, University of New South Wales, Sydney, NSW, Australia

**Keywords:** mental health, well-being, adolescence, psychiatric disorders, developmental disorders, psychometric testing, reliability, validity

The **Conflict of interest** statement was erroneously given as:

“The intellectual property rights to the COMPAS-W scale are assigned to Tilt Your Life Pty Ltd, of which JG is Founder and Director. The scale is available for non-commercial academic and research use and is licensed for commercial use via Tilt Your Life Pty Ltd (contact: justine.gatt@gmail.com).

The remaining author(s) declare that the research was conducted in the absence of any commercial or financial relationships that could be construed as a potential conflict of interest.”

The updated **Conflict of interest** statement is:

“Since original publication, JG has founded Tilt Your Life Pty Ltd, a wellbeing-focused company.

The remaining author(s) declare that the research was conducted in the absence of any commercial or financial relationships that could be construed as a potential conflict of interest.”

The original version of this article has been updated.

