# Supplementary material for: Measuring mental wellbeing in clinical and non-clinical adolescents using the COMPAS-W Wellbeing Scale
Source: Front Psychiatry. 2024 Jun 27;15:1333828. doi: 10.3389/fpsyt.2024.1333828 (PMC11234841; doi:10.3389/fpsyt.2024.1333828)
Supplement: Supplementary file 1 [file DataSheet1.docx]

Supplementary Material for

Measuring Mental Wellbeing in Clinical and Non-clinical Adolescents using the COMPAS-W Wellbeing Scale

**Janine R. Lam^1,2^, Haeme R.P. Park^1,2^, Justine M. Gatt^1,2^***

^1^ Centre for Wellbeing, Resilience and Recovery, Neuroscience Research Australia, Sydney, NSW, Australia

^2^ School of Psychology, University of New South Wales, Sydney, NSW, Australia

***Corresponding Author:**
Justine M. Gatt

Neuroscience Research Australia

Margarete Ainsworth Building

Barker Street

Randwick 2031

Australia

Email: [j.gatt@unsw.edu.au](mailto:j.gatt@unsw.edu.au)

Phone: +61 2 9399 1812

**Table of Contents**

**Supplementary Table 1.** [The original 26-item COMPAS-W scale validated in adults is available in Gatt et al., 2014. 3](#_Toc229162878)

[**Supplementary Table 2. Total scale and sub-scale raw, z-scores, percentiles and score range for COMPAS-W (23 items) for the whole sample (non-clinical and clinical groups, n = 1,078), and the corresponding 26-item raw score equivalent for comparison (derived from previous adult samples; Gatt, 2014)** 4](#_Toc229162879)

[**Supplementary Table 3. COMPAS-W (23-item) scale cut-offs for whole sample and non-clinical ‘healthy’ participants** 12](#_Toc229162880)

[**Supplementary Results.** 13](#_Toc229162881)

### **Supplementary Table 1.**

### The original 26-item COMPAS-W scale validated in adults is available in Gatt et al., 2014. Further inquiries can be directed to the corresponding author.

### **Supplementary Table 2. Total scale and sub-scale raw, z-scores, percentiles and score range for COMPAS-W (23 items) for the whole sample (non-clinical and clinical groups, n = 1,078), and the corresponding 26-item raw score equivalent for comparison (derived from previous adult samples; Gatt, 2014)**

| **Scale** | **Raw score (23-item version)** | **Raw score**  **(26-item equivalent)** | **Z-score**  **(23-item version)** | **Percentile**  **(23-item version)** | **Score Range**  **(23-item version)** |
| --- | --- | --- | --- | --- | --- |
| **Total Wellbeing** | 29 | 32.7 | -4.58 | 0.09 | 23 to 115 |
|  | 34 | 38.4 | -4.19 | 0.19 |  |
|  | 37 | 41.8 | -3.96 | 0.28 |  |
|  | 40 | 45.2 | -3.72 | 0.37 |  |
|  | 44 | 49.7 | -3.42 | 0.56 |  |
|  | 46 | 51.8 | -3.27 | 0.65 |  |
|  | 46 | 52.0 | -3.26 | 0.83 |  |
|  | 48 | 54.3 | -3.11 | 0.93 |  |
|  | 49 | 55.4 | -3.03 | 1.02 |  |
|  | 51 | 57.7 | -2.88 | 1.11 |  |
|  | 52 | 58.8 | -2.80 | 1.21 |  |
|  | 53 | 59.8 | -2.73 | 1.30 |  |
|  | 53 | 59.9 | -2.72 | 1.39 |  |
|  | 54 | 61.0 | -2.65 | 1.48 |  |
|  | 56 | 63.3 | -2.49 | 1.58 |  |
|  | 57 | 64.4 | -2.41 | 1.95 |  |
|  | 58 | 65.6 | -2.34 | 2.13 |  |
|  | 59 | 66.7 | -2.26 | 2.23 |  |
|  | 59 | 66.7 | -2.26 | 2.32 |  |
|  | 59 | 66.8 | -2.26 | 2.41 |  |
|  | 60 | 67.8 | -2.18 | 2.78 |  |
|  | 61 | 69.0 | -2.11 | 3.25 |  |
|  | 62 | 70.1 | -2.03 | 3.43 |  |
|  | 63 | 71.2 | -1.95 | 3.80 |  |
|  | 64 | 72.3 | -1.88 | 4.08 |  |
|  | 64 | 72.4 | -1.87 | 4.17 |  |
|  | 65 | 73.4 | -1.80 | 4.27 |  |
|  | 65 | 73.5 | -1.80 | 5.29 |  |
|  | 66 | 74.4 | -1.73 | 5.38 |  |
|  | 66 | 74.6 | -1.72 | 5.47 |  |
|  | 67 | 75.7 | -1.64 | 6.03 |  |
|  | 68 | 76.5 | -1.59 | 6.12 |  |
|  | 68 | 76.8 | -1.57 | 6.22 |  |
|  | 68 | 76.9 | -1.57 | 6.86 |  |
|  | 69 | 77.5 | -1.53 | 6.96 |  |
|  | 69 | 77.9 | -1.50 | 7.05 |  |
|  | 69 | 78.0 | -1.49 | 8.07 |  |
|  | 70 | 78.9 | -1.43 | 8.16 |  |
|  | 70 | 79.1 | -1.41 | 9.00 |  |
|  | 70 | 79.7 | -1.38 | 9.09 |  |
|  | 71 | 80.1 | -1.35 | 9.18 |  |
|  | 71 | 80.3 | -1.34 | 10.02 |  |
|  | 72 | 81.3 | -1.27 | 10.11 |  |
|  | 72 | 81.4 | -1.26 | 10.76 |  |
|  | 72 | 81.7 | -1.24 | 10.85 |  |
|  | 72 | 81.8 | -1.23 | 10.95 |  |
|  | 73 | 82.5 | -1.18 | 11.04 |  |
|  | 73 | 82.5 | -1.18 | 12.52 |  |
|  | 74 | 83.4 | -1.12 | 12.62 |  |
|  | 74 | 83.6 | -1.11 | 12.80 |  |
|  | 74 | 83.7 | -1.10 | 13.91 |  |
|  | 74 | 83.7 | -1.10 | 14.10 |  |
|  | 75 | 84.6 | -1.04 | 14.19 |  |
|  | 75 | 84.7 | -1.03 | 14.29 |  |
|  | 75 | 84.8 | -1.03 | 14.38 |  |
|  | 75 | 84.8 | -1.03 | 15.49 |  |
|  | 76 | 85.5 | -0.98 | 15.58 |  |
|  | 76 | 85.8 | -0.96 | 15.68 |  |
|  | 76 | 85.9 | -0.95 | 16.70 |  |
|  | 76 | 86.1 | -0.94 | 16.79 |  |
|  | 77 | 86.7 | -0.90 | 16.88 |  |
|  | 77 | 87.0 | -0.87 | 18.27 |  |
|  | 77 | 87.1 | -0.87 | 18.37 |  |
|  | 78 | 88.1 | -0.80 | 18.46 |  |
|  | 78 | 88.2 | -0.80 | 19.94 |  |
|  | 78 | 88.7 | -0.76 | 20.04 |  |
|  | 79 | 89.0 | -0.74 | 20.13 |  |
|  | 79 | 89.3 | -0.72 | 21.34 |  |
|  | 80 | 90.0 | -0.67 | 21.43 |  |
|  | 80 | 90.4 | -0.64 | 23.10 |  |
|  | 80 | 90.5 | -0.64 | 23.19 |  |
|  | 80 | 90.5 | -0.64 | 23.28 |  |
|  | 80 | 91.0 | -0.61 | 23.38 |  |
|  | 81 | 91.5 | -0.57 | 23.65 |  |
|  | 81 | 91.5 | -0.57 | 23.75 |  |
|  | 81 | 91.6 | -0.57 | 25.97 |  |
|  | 82 | 92.6 | -0.50 | 26.07 |  |
|  | 82 | 92.7 | -0.49 | 26.16 |  |
|  | 82 | 92.7 | -0.49 | 28.66 |  |
|  | 82 | 92.8 | -0.48 | 28.76 |  |
|  | 83 | 93.5 | -0.43 | 28.85 |  |
|  | 83 | 93.7 | -0.42 | 28.94 |  |
|  | 83 | 93.8 | -0.41 | 30.80 |  |
|  | 83 | 94.3 | -0.38 | 30.89 |  |
|  | 84 | 95.0 | -0.33 | 32.84 |  |
|  | 84 | 95.5 | -0.30 | 32.93 |  |
|  | 85 | 95.8 | -0.28 | 33.02 |  |
|  | 85 | 96.0 | -0.26 | 33.12 |  |
|  | 85 | 96.0 | -0.26 | 33.21 |  |
|  | 85 | 96.1 | -0.26 | 33.30 |  |
|  | 85 | 96.1 | -0.26 | 33.40 |  |
|  | 85 | 96.1 | -0.26 | 35.71 |  |
|  | 85 | 96.1 | -0.26 | 35.81 |  |
|  | 86 | 97.1 | -0.19 | 35.90 |  |
|  | 86 | 97.1 | -0.19 | 35.99 |  |
|  | 86 | 97.1 | -0.19 | 36.09 |  |
|  | 86 | 97.2 | -0.18 | 39.15 |  |
|  | 86 | 97.3 | -0.18 | 39.24 |  |
|  | 86 | 97.3 | -0.17 | 39.33 |  |
|  | 86 | 97.4 | -0.17 | 39.42 |  |
|  | 86 | 97.7 | -0.15 | 39.52 |  |
|  | 87 | 98.2 | -0.11 | 39.80 |  |
|  | 87 | 98.3 | -0.10 | 43.41 |  |
|  | 87 | 98.4 | -0.10 | 43.51 |  |
|  | 87 | 98.4 | -0.10 | 43.60 |  |
|  | 87 | 98.7 | -0.08 | 43.69 |  |
|  | 88 | 99.1 | -0.05 | 43.78 |  |
|  | 88 | 99.3 | -0.04 | 43.88 |  |
|  | 88 | 99.4 | -0.03 | 43.97 |  |
|  | 88 | 99.5 | -0.03 | 46.85 |  |
|  | 88 | 99.9 | 0.00 | 46.94 |  |
|  | 88 | 100.0 | 0.01 | 47.03 |  |
|  | 89 | 100.3 | 0.03 | 47.12 |  |
|  | 89 | 100.4 | 0.04 | 47.22 |  |
|  | 89 | 100.5 | 0.04 | 47.31 |  |
|  | 89 | 100.5 | 0.05 | 47.40 |  |
|  | 89 | 100.6 | 0.05 | 49.81 |  |
|  | 89 | 100.7 | 0.05 | 49.91 |  |
|  | 89 | 100.8 | 0.07 | 50.00 |  |
|  | 89 | 101.1 | 0.09 | 50.09 |  |
|  | 90 | 101.2 | 0.09 | 50.19 |  |
|  | 90 | 101.3 | 0.10 | 50.28 |  |
|  | 90 | 101.4 | 0.11 | 50.37 |  |
|  | 90 | 101.6 | 0.12 | 50.46 |  |
|  | 90 | 101.6 | 0.12 | 50.93 |  |
|  | 90 | 101.7 | 0.13 | 54.27 |  |
|  | 90 | 101.7 | 0.13 | 54.36 |  |
|  | 91 | 102.7 | 0.19 | 54.45 |  |
|  | 91 | 102.8 | 0.20 | 54.64 |  |
|  | 91 | 102.9 | 0.21 | 57.70 |  |
|  | 91 | 103.4 | 0.24 | 57.79 |  |
|  | 92 | 103.9 | 0.28 | 57.98 |  |
|  | 92 | 104.0 | 0.28 | 61.69 |  |
|  | 92 | 104.1 | 0.29 | 61.87 |  |
|  | 92 | 104.1 | 0.29 | 61.97 |  |
|  | 92 | 104.4 | 0.31 | 62.06 |  |
|  | 93 | 105.0 | 0.35 | 62.15 |  |
|  | 93 | 105.1 | 0.36 | 64.66 |  |
|  | 93 | 105.1 | 0.36 | 64.75 |  |
|  | 94 | 105.9 | 0.41 | 64.84 |  |
|  | 94 | 106.1 | 0.42 | 64.94 |  |
|  | 94 | 106.2 | 0.43 | 65.12 |  |
|  | 94 | 106.2 | 0.43 | 65.21 |  |
|  | 94 | 106.3 | 0.44 | 68.37 |  |
|  | 94 | 106.3 | 0.44 | 68.46 |  |
|  | 95 | 106.8 | 0.48 | 68.55 |  |
|  | 95 | 107.3 | 0.50 | 68.65 |  |
|  | 95 | 107.3 | 0.51 | 68.74 |  |
|  | 95 | 107.4 | 0.51 | 70.96 |  |
|  | 95 | 107.4 | 0.52 | 71.06 |  |
|  | 96 | 108.1 | 0.56 | 71.15 |  |
|  | 96 | 108.2 | 0.57 | 71.24 |  |
|  | 96 | 108.4 | 0.58 | 71.34 |  |
|  | 96 | 108.5 | 0.59 | 74.03 |  |
|  | 96 | 108.6 | 0.59 | 74.12 |  |
|  | 97 | 109.6 | 0.66 | 74.30 |  |
|  | 97 | 109.7 | 0.67 | 76.44 |  |
|  | 98 | 110.3 | 0.71 | 76.53 |  |
|  | 98 | 110.7 | 0.74 | 76.62 |  |
|  | 98 | 110.8 | 0.74 | 78.76 |  |
|  | 98 | 110.8 | 0.75 | 78.85 |  |
|  | 99 | 111.6 | 0.80 | 78.94 |  |
|  | 99 | 111.9 | 0.82 | 80.80 |  |
|  | 100 | 112.9 | 0.89 | 80.89 |  |
|  | 100 | 113.0 | 0.89 | 80.98 |  |
|  | 100 | 113.0 | 0.90 | 83.12 |  |
|  | 101 | 114.0 | 0.96 | 83.21 |  |
|  | 101 | 114.1 | 0.97 | 83.30 |  |
|  | 101 | 114.1 | 0.97 | 83.40 |  |
|  | 101 | 114.2 | 0.98 | 84.69 |  |
|  | 101 | 114.2 | 0.98 | 84.97 |  |
|  | 101 | 114.6 | 1.00 | 85.06 |  |
|  | 102 | 114.9 | 1.02 | 85.16 |  |
|  | 102 | 115.3 | 1.05 | 86.92 |  |
|  | 102 | 115.8 | 1.08 | 87.01 |  |
|  | 103 | 116.1 | 1.10 | 87.11 |  |
|  | 103 | 116.4 | 1.13 | 88.96 |  |
|  | 103 | 116.8 | 1.16 | 89.05 |  |
|  | 104 | 117.6 | 1.21 | 90.54 |  |
|  | 105 | 118.6 | 1.28 | 90.63 |  |
|  | 105 | 118.6 | 1.28 | 90.72 |  |
|  | 105 | 118.7 | 1.28 | 91.74 |  |
|  | 105 | 119.2 | 1.32 | 91.84 |  |
|  | 106 | 119.6 | 1.34 | 91.93 |  |
|  | 106 | 119.8 | 1.36 | 93.23 |  |
|  | 107 | 120.9 | 1.43 | 93.32 |  |
|  | 107 | 121.0 | 1.44 | 94.34 |  |
|  | 108 | 121.7 | 1.49 | 94.43 |  |
|  | 108 | 122.0 | 1.51 | 94.53 |  |
|  | 108 | 122.1 | 1.51 | 95.73 |  |
|  | 109 | 123.1 | 1.58 | 95.83 |  |
|  | 109 | 123.2 | 1.59 | 96.29 |  |
|  | 110 | 124.2 | 1.66 | 96.38 |  |
|  | 110 | 124.3 | 1.67 | 97.03 |  |
|  | 111 | 125.3 | 1.74 | 97.12 |  |
|  | 111 | 125.4 | 1.74 | 97.22 |  |
|  | 111 | 125.5 | 1.75 | 97.77 |  |
|  | 112 | 126.6 | 1.82 | 98.33 |  |
|  | 113 | 127.7 | 1.90 | 98.61 |  |
|  | 114 | 128.9 | 1.98 | 99.17 |  |
|  | 115 | 130.0 | 2.05 | 100.00 |  |
|  |  |  |  |  |  |
| **COMPOSURE** | 4 | 4.0 | -3.35 | 0.68 | 4 to 20 |
|  | 5 | 5.0 | -3.03 | 0.77 |  |
|  | 6 | 6.0 | -2.72 | 1.35 |  |
|  | 7 | 7.0 | -2.40 | 2.32 |  |
|  | 8 | 8.0 | -2.08 | 3.96 |  |
|  | 9 | 9.0 | -1.77 | 5.69 |  |
|  | 10 | 10.0 | -1.45 | 9.85 |  |
|  | 11 | 11.0 | -1.13 | 15.44 |  |
|  | 12 | 12.0 | -0.82 | 25.29 |  |
|  | 13 | 13.0 | -0.50 | 33.69 |  |
|  | 14 | 14.0 | -0.18 | 47.01 |  |
|  | 15 | 15.0 | 0.13 | 58.30 |  |
|  | 16 | 16.0 | 0.45 | 71.43 |  |
|  | 17 | 17.0 | 0.76 | 82.14 |  |
|  | 18 | 18.0 | 1.08 | 89.29 |  |
|  | 19 | 19.0 | 1.40 | 94.40 |  |
|  | 20 | 20.0 | 1.71 | 100.00 |  |
|  |  |  |  |  |  |
| **OWN-WORTH** | 4 | 9.0 | -3.57 | 0.10 | 4 to 20 |
|  | 5 | 11.3 | -3.24 | 0.19 |  |
|  | 6 | 13.5 | -2.90 | 0.76 |  |
|  | 7 | 15.8 | -2.56 | 1.62 |  |
|  | 8 | 18.0 | -2.22 | 2.67 |  |
|  | 9 | 20.3 | -1.89 | 4.39 |  |
|  | 10 | 22.5 | -1.55 | 7.15 |  |
|  | 11 | 24.8 | -1.21 | 14.39 |  |
|  | 12 | 27.0 | -0.87 | 25.74 |  |
|  | 13 | 29.3 | -0.53 | 36.80 |  |
|  | 14 | 31.5 | -0.20 | 47.00 |  |
|  | 15 | 33.8 | 0.14 | 59.20 |  |
|  | 16 | 36.0 | 0.48 | 73.21 |  |
|  | 17 | 38.3 | 0.82 | 82.55 |  |
|  | 18 | 40.5 | 1.15 | 90.28 |  |
|  | 19 | 42.8 | 1.49 | 95.52 |  |
|  | 20 | 45.0 | 1.83 | 100.00 |  |
|  |  |  |  |  |  |
| **MASTERY** | 4 | 6.0 | -4.40 | 0.10 | 4 to 20 |
|  | 5 | 7.5 | -4.02 | 0.19 |  |
|  | 6 | 9.0 | -3.65 | 0.48 |  |
|  | 8 | 12.0 | -2.89 | 1.15 |  |
|  | 9 | 13.5 | -2.51 | 2.11 |  |
|  | 10 | 15.0 | -2.13 | 3.54 |  |
|  | 11 | 16.5 | -1.75 | 6.32 |  |
|  | 12 | 18.0 | -1.37 | 12.25 |  |
|  | 13 | 19.5 | -0.99 | 19.71 |  |
|  | 14 | 21.0 | -0.62 | 29.76 |  |
|  | 15 | 22.5 | -0.24 | 42.78 |  |
|  | 16 | 24.0 | 0.14 | 63.92 |  |
|  | 17 | 25.5 | 0.52 | 76.27 |  |
|  | 18 | 27.0 | 0.90 | 85.55 |  |
|  | 19 | 28.5 | 1.28 | 92.73 |  |
|  | 20 | 30.0 | 1.66 | 100.00 |  |
|  |  |  |  |  |  |
| **POSITIVITY** | 6 | 6.0 | -4.13 | 0.2 | 5 to 25 |
|  | 7 | 7.0 | -3.82 | 0.5 |  |
|  | 8 | 8.0 | -3.52 | 0.6 |  |
|  | 9 | 9.0 | -3.21 | 0.8 |  |
|  | 10 | 10.0 | -2.90 | 1.5 |  |
|  | 11 | 11.0 | -2.60 | 2.4 |  |
|  | 12 | 12.0 | -2.29 | 3.9 |  |
|  | 13 | 13.0 | -1.98 | 4.6 |  |
|  | 14 | 14.0 | -1.67 | 6.9 |  |
|  | 15 | 15.0 | -1.37 | 11.3 |  |
|  | 16 | 16.0 | -1.06 | 14.3 |  |
|  | 17 | 17.0 | -0.75 | 21.4 |  |
|  | 18 | 18.0 | -0.45 | 32.7 |  |
|  | 19 | 19.0 | -0.14 | 45.7 |  |
|  | 20 | 20.0 | 0.17 | 63.2 |  |
|  | 21 | 21.0 | 0.48 | 75.9 |  |
|  | 22 | 22.0 | 0.78 | 83.6 |  |
|  | 23 | 23.0 | 1.09 | 88.3 |  |
|  | 24 | 24.0 | 1.40 | 94.4 |  |
|  | 25 | 25.0 | 1.70 | 100 |  |
|  |  |  |  |  |  |
| **ACHIEVEMENT** | 3 | 3.0 | -3.76 | 0.38 | 3 to 15 |
|  | 5 | 5.0 | -2.90 | 0.95 |  |
|  | 6 | 6.0 | -2.47 | 2.86 |  |
|  | 7 | 7.0 | -2.05 | 4.77 |  |
|  | 8 | 8.0 | -1.62 | 8.30 |  |
|  | 9 | 9.0 | -1.19 | 16.70 |  |
|  | 10 | 10.0 | -0.76 | 27.10 |  |
|  | 11 | 11.0 | -0.34 | 38.17 |  |
|  | 12 | 12.0 | 0.09 | 62.88 |  |
|  | 13 | 13.0 | 0.52 | 74.14 |  |
|  | 14 | 14.0 | 0.95 | 84.73 |  |
|  | 15 | 15.0 | 1.37 | 100.00 |  |
|  |  |  |  |  |  |
| **SATISFACTION** | 7 | 9.0 | -3.97 | 0.30 | 7 to 35 |
|  | 8 | 10.3 | -3.78 | 0.40 |  |
|  | 9 | 11.6 | -3.59 | 0.60 |  |
|  | 11 | 14.1 | -3.20 | 0.81 |  |
|  | 12 | 15.4 | -3.01 | 1.21 |  |
|  | 13 | 16.7 | -2.81 | 1.41 |  |
|  | 14 | 18.0 | -2.62 | 1.81 |  |
|  | 15 | 19.3 | -2.43 | 2.32 |  |
|  | 16 | 20.6 | -2.23 | 3.22 |  |
|  | 17 | 21.9 | -2.04 | 4.13 |  |
|  | 18 | 23.1 | -1.85 | 5.34 |  |
|  | 19 | 24.4 | -1.65 | 7.35 |  |
|  | 20 | 25.7 | -1.46 | 9.16 |  |
|  | 21 | 27.0 | -1.27 | 12.89 |  |
|  | 22 | 28.3 | -1.07 | 16.31 |  |
|  | 23 | 29.6 | -0.88 | 19.54 |  |
|  | 24 | 30.9 | -0.69 | 24.17 |  |
|  | 25 | 32.1 | -0.49 | 28.90 |  |
|  | 26 | 33.4 | -0.30 | 37.26 |  |
|  | 27 | 34.7 | -0.11 | 43.50 |  |
|  | 28 | 36.0 | 0.09 | 55.39 |  |
|  | 29 | 37.3 | 0.28 | 62.44 |  |
|  | 30 | 38.6 | 0.47 | 68.88 |  |
|  | 31 | 39.9 | 0.67 | 75.83 |  |
|  | 32 | 41.1 | 0.86 | 82.07 |  |
|  | 33 | 42.4 | 1.05 | 87.21 |  |
|  | 34 | 43.7 | 1.25 | 91.54 |  |
|  | 35 | 45.0 | 1.44 | 100.00 |  |

### **Supplementary Table 3. COMPAS-W (23-item) scale cut-offs for whole sample and non-clinical ‘healthy’ participants**

| **COMPAS-W Scale**  **(23-item adolescent version)** | **Whole sample**  **(clinical and non-clinical)** | | **Non-clinical sample only (healthy)** | |
| --- | --- | --- | --- | --- |
|  | **Raw score cut-offs** | **Group category** | **Raw score cut-offs** | **Group category** |
| Total Wellbeing | <75  76-100  101+ | Languishing  Moderate  Flourishing | <78  79-101  102+ | Languishing  Moderate  Flourishing |
| Composure | <11  12-17  18+ | Languishing  Moderate  Flourishing | <12  13-17  18+ | Languishing  Moderate  Flourishing |
| Own-worth | <11  12-17  18+ | Languishing  Moderate  Flourishing | <12  13-17  18+ | Languishing  Moderate  Flourishing |
| Mastery | <13  14-18  19+ | Languishing  Moderate  Flourishing | <13  14-18  19+ | Languishing  Moderate  Flourishing |
| Positivity | <16  17-22  23+ | Languishing  Moderate  Flourishing | <17  18-22  23+ | Languishing  Moderate  Flourishing |
| Achievement | <9  10-13  14+ | Languishing  Moderate  Flourishing | <10  11-13  14+ | Languishing  Moderate  Flourishing |
| Satisfaction | <22  23-32  33+ | Languishing  Moderate  Flourishing | <23  24-32  33+ | Languishing  Moderate  Flourishing |

*Note.* This table shows the raw score cut-offs that can be used for categorisation scoring into ‘Languishing’, ‘Moderate’, and ‘Flourishing’ groups based on the general population sample (whole sample) (n=1078) or when recalculated on the non-clinical (healthy) subsample (n=853) alone. For both cases, they are based on z-score cut-offs of -1 and +1. These cut-offs are based on the averages and variances of these samples, and so should be interpreted as an approximate guide only.

### **Supplementary Results.**

In addition to Cronbach alpha reliability (presented in main manuscript), ‘composite reliability’ (which takes into consideration the factor loading weights in the CFA model) was also calculated for the original 26-item base model and the final 23-item model for comparison. For the 26-item base model, the estimates were as follows: Total scale, *CR* = 0.871, Composure, *CR* = 0.504; Own-worth, *CR* = 0.440; Mastery, *CR* = 0.797; Positivity, *CR* = 0.658; Achievement, *CR* = 0.830; and Satisfaction, *CR* = 0.933). For the new 23-item COMPAS-W model, the estimates were as follows: Total scale, *CR* = 0.894, Composure, *CR* = 0.557; Own-worth, *CR* = 0.536; Mastery, *CR* = 0.761; Positivity, *CR* = 0.690; Achievement, *CR* = 0.830; and Satisfaction, *CR* = 0.833. Therefore, increases in the estimates were observed for the Total scale, Composure, Own-worth, and Positivity scales, while estimates for Achievement remained the same, and estimates for Mastery and Satisfaction reduced. Again, we caution against citing these estimates when comparing estimates of this scale to other wellbeing scales in the literature as these estimates are often not calculated or reported in other studies so the comparisons are not equivalent. Henceforth, we focus on the Cronbach alpha results.
